# Synopsis of *Phyllosticta* in China

**DOI:** 10.1080/21501203.2015.1027507

**Published:** 2015-04-17

**Authors:** Ke Zhang, Roger G. Shivas, Lei Cai

**Affiliations:** a State Key Laboratory of Mycology, Institute of Microbiology, Chinese Academy of Sciences, Beijing100101, P. R. China; b Plant Pathology Herbarium, Biosecurity Queensland, Ecosciences Precinct, Dutton Park, QLD4102, Australia

**Keywords:** *Guignardia*, inventory, phytopathogenic fungi, systematics, taxonomy

## Abstract

The generic concept of *Phyllosticta* has undergone substantial changes since its establishment in 1818. The existence of conidia with a mucilaginous sheath and an apical appendage is synapomorphic for *Phyllosticta* species, which has been shown in recent molecular phylogenetic studies. Thus a natural classification of *Phyllosticta* species should emphasize above morphological characters. Many names in *Phyllosticta*, both published in the scientific literatures and in publically accessible databases, need updating. In China, more than 200 species names in *Phyllosticta* have been recorded, of which, 158 species names are reviewed here based on their morphological descriptions and molecular data. Only 20 species of *Phyllosticta* are accepted from China. Other records of *Phyllosticta* refer to *Phoma* (89 records), *Asteromella* (14 records), *Boeremia* (9 records), *Phomopsis* (7 records) and *Microsphaeropsis* (1 record), with 19 names of uncertain generic classification. This work demonstrates an urgent need for the re-assessment of records of *Phyllosticta* worldwide.

## Introduction

*Phyllosticta* is an important group of plant pathogenic fungi distributed worldwide that cause serious diseases, e.g. citrus black spot (Baayen et al. 2002), cranberry early rot (Shear 1907). Many records of *Phyllosticta* appear in the literature under the later synonym *Guignardia*, which was introduced for the teleomorphic stage of the fungus (van der Aa 1973; Hyde 1995; Crous et al. 1996; Wulandari et al. 2010).

Since the genus *Phyllosticta* was established by Persoon (1818), more than 3000 species’ epithets have been recorded. Early classification of *Phyllosticta* was mostly based on host association and disease symptoms (Desmazières 1847; Saccardo 1884). Desmazières (1847) revised *Phyllosticta*, and considered it was characterized as leaf spotting fungi that had small conidia and globose pycnidia (Desmazières 1847). Saccardo (1878, 1884) later defined *Phyllosticta* as a group of fungi parasitic on leaves, with 1-celled, ovoid or oblong, hyaline conidia. Unfortunately, many morphologically similar fungi occur on leaf spots, e.g. *Asteromella, Phoma* or *Phomopsis*, which has led to much confusion in the plant pathological and mycological literature.

The most recent morphology-based revisionary treatments define *Phyllosticta* (van der Aa 1973; van der Aa and Vanev 2002) as having globose, subglobose or tympaniform pycnidia: 1-celled, globose, subglobose, ellipsoidal, ovoidal, obovoidal or pyriform conidia with slime sheaths and an apical appendage. van der Aa and Vanev (2002) considered more than 2000 species names, accepting only 141 species. The existence of conidia with a mucilaginous sheath and an apical appendage is synapomorphic for *Phyllosticta* species in recent molecular phylogenetic studies (Motohashi et al. 2009; Wikee et al. 2011; Su and Cai 2012; Zhang, Su, et al. 2013). Recent molecular studies have portrayed clearer phylogenetic relationships in the group, based on DNA sequence analysis of conconcatenated intron-dominated genes such as ITS, ACT, TEF, and highly conserved gene coding regions such as LSU and GAPDH. These studies have recognized many cryptic species in traditionally morphologically circumscribed species complexes, e.g. *P. citricarpa* on citrus, *P. musarum* on banana, *P. vaccinii* on *Vaccinium, G. philoprina* on *Rhododendron, Hedera, Ilex, Magnolia* and *Taxus* (Wulandari et al. 2009; Glienke et al. 2011; Wang et al. 2012; Wikee et al. 2013; Zhang, Zhang, et al. 2013).

The morphological characters of *Phoma* are: pycnidia immersed, globose to subglobose or obpyriform, pale brown to dark brown, with one central ostiole, sometimes with more than one ostiole in culture; conidiogenous cells hyaline, short, phialidic, enteroblastic, obpyriform, formed from the cells lining the inside of the pycnidium; conidia hyaline, 1-celled, occasionally 1-septate, usually biguttulate, oblong, obovate or ellipsoidal, asepta conidia usually 3–12 μm long, conidia with 1–3 septa usually 8–15 μm long, 0.5–5 μm wide; chlamydospores are often formed in culture (van der Aa and Vanev 2002; Boerema et al. 2004).

The morphological characters of *Asteromella* are: conidiomata pycnidial, globose, separate or more frequently aggregated, dark brown, immersed, unilocular, thick-walled, wall of brown; ostiole central, circular, papillate; conidiophores hyaline, smooth, 1–3 septate, tapered at the apex, branched only at the base, formed from the inner cells of the pycnidial wall; conidiogenous cells enteroblastic, phialidic, integrated or less often discrete, determinate, hyaline, apertures apical or lateral on short branches produced immediately below transverse sept; conidia hyaline, aseptate, thin-walled, eguttulate, cylindrical to oval (Sutton 1980; van der Aa and Vanev 2002).

The morphological characters of *Phomopsis* are: pycnidia, brown to black, scattered or aggregated, globose to conical, convulated to unilocular, singly ostiolate, pycnidial wall consisting of brown; conidiophores hyaline, cylindrical, branched above or below, 1–3-septate; conidiogenous cells straight to curved, tapering slightly towards the apex. α-conidia 1-celled, hyaline, fusoid to ellipsoidal, apex bluntly rounded, biguttulate; β-conidia 1-celled, hyaline, filamentous, usually curved at upper half, without guttules (Muntaňola-Cvetcovic et al. 1981; van der Aa and Vanev 2002).

The first report of *Phyllosticta* in China (Miura 1928) listed 24 species from northeast China. Since then, more than 200 records of *Phyllosticta* have been reported from China by Teng (1963), Tai (1979) and subsequent mycologists. Most of these records were reported and described in the *Flora Fungorum Sinicorm,* Vol. 15 (Bai 2003). Bai (2003) accepted the significance of the slime sheath and apical appendage in identifying *Phyllosticta* species, but he listed many species that did not possess these structures under *Phyllosticta*. More recently, some newly described species have been reported from China (Su and Cai 2012; Zhang, Su, et al. 2013). An updated checklist of *Phyllosticta* species in China is needed, especially as some of these species have biosecurity significance.

We examined 158 records of *Phyllosticta* recorded in *Flora Fungorum Sinicorm* Vol. 15 and published subsequently. Earlier records were not considered. Most of the recorded species were reclassified based on morphology, while the generic identity of 19 records could not be determined. Of the remaining 139 records of *Phyllosticta*, 89 were considered to be *Phoma*, 14 *Asteromella*, 9 *Boeremia*, 7 *Phomopsis* and 1 *Microsphaeropsis*. Only 20 species actually belonged to *Phyllosticta*. Notes for each species and a check list of accepted *Phyllosticta* species names in China are provided (Table 1).

**Table 1.** Check list of accepted *Phyllosticta* species in China.

Species	Host	Conidiogenous cells	Conidia	References
*P. amaryllidicola*	*Lycoris radiata*	4–6 × 2–4 μm, ampulliform, 1-celled, hyaline	9–11 × 6–7 μm, ovoid, ellipsoidal, 1-celled, hyaline	Bai (2003)
*P. arecae*	*Areca catechu*	7.5–10 × 5–6 μm, ampulliform, 1-celled, hyaline	6–12.5 × 5–7 μm, ovoid, ellipsoidal, cylindrical, 1-celled, hyaline	Bai (2003)
*P. capitalensis*	Various	7–20 × 3–7 μm, subcylindrical to ampulliform, frequently reduced to conidiogenous cells, or branching from a basal supporting cell	7–10 × 3–5 μm, terminal, subcylindrical to ampulliform to doliiform, hyaline, smooth	Glienke et al. (2011)
*P. carochlae*	*Caryota ochlandra*	2–6.5 × 5.5–13 μm, holoblastic, hyaline, cylindrical, proliferating 1–2 times percurrently	6–8.5 × 10–12 μm, unicellular, ovoid, obovoid, ellipsoidal to subglobose, enclosed in a mucilaginous sheath, and bearing a hyaline, mucoid apical appendage	Zhou et al. (2015)
*P. citriasiana*	*Citrus maxima*	7–17 × 3–5 μm, terminal, subcylindrical to ampulliform or somewhat doliiform, hyaline, smooth	12–14 × 6–7 μm, solitary, hyaline, aseptate, thin- and smooth-walled, coarsely guttulate, ellipsoidal to obovoid	Wulandari et al. (2009)
*P. citricarpa*	*Citrus* spp.	7–12 × 3–4 μm, terminal, subcylindrical to somewhat doliiform, hyaline, smooth	11–12 × 7 μm, solitary, hyaline, aseptate, thin- and smooth-walled, coarsely guttulate, ellipsoid to obovoid, tapering toward a narrowly truncate base	Glienke et al. (2011)
*P. citrichinaensis*	*Citrus* spp.	6–12 × 2–5 μm, holoblastic, phialidic, short cylindrical, lageniform, unicellular, thinwalled, smooth	8–12 × 6–9 μm, ellipsoid to ovoid, with rounded ends, unicellular, thin, smooth-walled, pomiform, colourless	Wang et al. (2012)
*P. cruenta*	*Polygonatum macropodium*	8–15 × 2–5 μm, ampulliform, cylindrical 1-celled, hyaline	10–14 × 7–8 μm, ovoid, 1-celled, hyaline	Bai (2003)
*P. euonymi-japonici*	*Euonymus japonicus*	15–22.5 × 3.75–5 μm, holoblastic, phialidic, short cylindrical, lageniform, unicellular, colourless	10–12.5 × 7.5–10 μm, ellipsoid to ovoid, hyaline, with rounded ends, unicellular, thin, smooth-walled	Liu and Lu (2007)
*P. ghaesembillae*	*Codiaeum variegatum*	4–6 × 2–3.5 μm, ampulliform, 1-celled, hyaline	10–13 × 7–7.5 μm, ovoid, 1-celled, hyaline	Bai (2003)
*P. hemerocallidis*	*Hemerocallis fulva*	10–12.5 × 5 μm, ampulliform, 1-celled, hyaline	7.5–12.5 × 5–7 μm, ovoid, ellipsoidal, 1-celled, hyaline	Bai (2003)
*P. hostae*	*Hosta plantaginea*	7–22 × 2–5 μm, holoblastic, phialidic, cylindrical, subcylindrical to ampulliform, hyaline, thin-walled, smooth	8–15 × 5–9 μm, unicellular, thin- and smooth-walled, ellipsoid, subglobose to obovoid, truncate at the base when young, later rounded at both ends	Su and Cai (2012)
*P. hubeiensis*	*Viburnum odoratissimum*	6–12.5 × 3–5 μm, holoblastic, hyaline, cylindrical or conical	10–14.5 × 6–9 μm, 1-celled, ellipsoid to obovoid, truncate at the base sometimes	Zhang, Su, et al. (2013)
*P. minima*	*Acer cinnamomifolium*	4–7.5 × 3–7 μm, ampulliform, 1-celled, hyaline	7.5–12 × 4–7 μm, ovoid, ellipsoidal, 1-celled, hyaline	Bai (2003)
*P. murrayicola*	*Murraya paniculata*	4–6 × 2–3.5 μm, ampulliform, 1-celled, hyaline	10–12.5 × 6–8 μm, ovoid, pyriform, 1-celled, hyaline	Bai (2003)
*P. musaechinensis*	*Musa* sp.	cylindrical or conical	14–18 × 8–12 μm, hyaline, aseptate, coarsely guttulate, ellipsoidal or clavate, thin- and smooth-walled, surrounded by a mucilaginous sheath, apex tapering, straight to curved, appendage 4.0–18.5 μm long	Wu et al. (2014)
*P. musarum*	*Musa* sp.	–	15–18 × 9–10 µm, 1-celled, obovoidal, ellipsoidal or short cylindrical, pyriform when young, with a truncate base, broadly rounded	Pu et al. (2008)
*P. schimae*	*Schima superba*	8–30 × 2–4 μm, holoblastic, phialidic, short cylindrical, subcylindrical to ampulliform, hyaline, thin-walled, smooth	7–13 × 4–7 μm, unicellular, thin- and smooth-walled, globose, ellipsoid to obovoid, truncate at the base when young, later rounded at both ends	Su and Cai (2012)
*P. schimicola*	*Schima superba*	1.5–4.5 × 5–12 μm, holoblastic, hyaline, long cylindrical, subcylindrical to ampulliform, proliferating 1–2 times percurrently	5–8 × 8–11 μm, unicellular, smooth-walled, ovoid to long ovoid, ampulliform, ellipsoidal to subglobose, truncate at the base when young, enclosed in a smooth and a mucilaginous sheath, and bearing a hyaline, mucoid apical appendage.	Zhou et al. (2015)
*P. styracicola*	*Styrax grandiflorus*	2–3.5 × 8–12.5 μm, holoblastic, hyaline, cylindrical	9.5–13 × 6.5–9 μm, 1-celled, ellipsoidal to subglobose	Zhang, Su, et al. (2013)

## Taxonomy

1. *Phyllosticta abutilonis* P. Hennings, Hedwigia 48: 13. 1908.

→ *Boeremia exigua* (Desm.) Aveskamp, Gruyter & Verkley.

Host: *Abutilon theophrasti* (Malvaceae).

Distribution: Inner Mongolia, Jilin, Liaoning.

Specimens: HMSAU 653, HMSAU 654, HMSAU 1398, HMSAU 1995.

Notes: The Chinese specimens had cylindrical, ellipsoidal and subglobose conidia that measured 56 × 23 μm, and lacked a mucilaginous sheath and an apical appendage (Bai 2003, p. 136). These characters are clearly atypical for *Phyllosticta* but are essentially similar to *Boeremia exigua* (Aveskamp et al. 2010).

2. *Phyllosticta aceris* Saccardo, Michelia 1: 147. 1878.

→ *Phoma* sp.

Host: *Acer saccharum, A. negundo* (Aceraceae).

Distribution: Jilin, Liaoning.

Specimens: HMSAU 1210, HMSAU 1174, HMSAU 2049, HMSAU 2076.

Notes: Many species of *Phyllosticta* have been reported from *Acer* spp., although only two, *P. capitalensis* and *P. minima*, were accepted by van der Aa and Vanev (2002). The Chinese specimens had oval or elliptical, 1-celled, hyaline, conidia with one guttule that measured 5–7.5 × 2–3.5 μm, seldom with short appendages (Bai 2003, p. 45).The conidial size is smaller than *P. capitalensis* (8–15 × 5–8 μm) and *P. minima* (7.5–12 × 4.5–8 μm) (van der Aa 1973). van der Aa and Vanev (2002) considered *Phyllosticta aceris* a *Phomopsis* species, as the conidia lacked a mucilaginous sheath and an apical appendage. The conidiogenous cells of the Chinese specimens were cylindrical, 1-celled, hyaline, 4–7.5 × 2–3 μm (Bai 2003, p. 45) which indicate that it belongs to *Phoma*.

3. *Phyllosticta acetosae* Saccardo, Michelia. 1: 151. 1878.

→ *Asteromella* sp. or *Phoma* sp.

Host: *Rumex* spp. (Rumiceae).

Distribution: Liaoning, Tibet.

Specimens: HMSAU 2718, HMSAU 2002, HMSAU 2003.

Notes: The conidia of *P. acetosae* were originally described as oblong or cylindrical, with 2 guttules, hyaline that measured 4–5 × 2 μm (Saccardo 1878). van der Aa and Vanev (2002) considered that this fungus belonged to *Asteromella*. The Chinese specimens had ampulliform conidiogenous cells, and 1-celled, ovoid to ellipsoidal hyaline conidia round at both ends that measured 3–5 × 2–2.5 μm, with 2 guttules (Bai 2003, p. 158), which also indicate an *Asteromella* or small-spored *Phoma* species.

4. *Phyllosticta acetosellae* Smith & Ramsbottom, Trans. Brit. Myc. Soc. 4 (1): 173. 1912 [1913].

→ *Phoma acetosellae* (A.L. Sm. & Ramsb.) van der Aa & Boerema.

Host: *Rumex* spp. (Rumiceae).

Distribution: Liaoning, Jilin, Tibet.

Specimens: HMSAU 1177, HMSAU 406, HMSAU 2685.

Notes: The Chinese specimens had conidia that are cylindrical, sometimes irregular, 1-celled, hyaline, biguttulate, 6–10 × 2–4 μm (Bai 2003, p. 159) and matched the description of *Phoma acetosellae* (van der Aa et al. 2002).

5. *Phyllosticta aglaiae* G.Z. Lu & J.K. Bai, in Yu et al., Journal of Shenyang Agricultural University, 25(2): 154, 1994.

→ *Asteromella* sp. or *Phoma* sp.

Host: *Aglaia odorata* (Meliaceae).

Distribution: Liaoning.

Specimen: HMSAU 2045.

Notes: When this species was first described, Yu et al. (1994) did not provide a description, so this name is invalid. The Chinese holotype had ovoid or ellipsoidal, 1-celled, hyaline, conidia with 2 guttules that measured 2.5–5 × 1–1.5 μm, and lacked a mucilaginous sheath and an apical appendage (Bai 2003, p. 139). This species does not belong to *Phyllosticta* but may be an *Asteromella* species or very small-spored *Phoma* species.

6. *Phyllosticta allescheri* Sydow, Hedwigia, 157, 1897.

→ *Phoma* sp.

Hosts: *Parthenocissus himalayana, Parthenocissus thunbergii* (Vitaceae).

Distribution: Jilin, Sichuan.

Specimens: HMSAU 2702, HMSAU 800.

Notes: The only *Phyllosticta* species described from *Parthenocissus* is *P. parthenocissisus* (Zhang, Zhang, et al. 2013), which was first classified as *P. ampelicida*. According to Sydow’s (1897) original description, the conidia of *P. allescheri* are 3–5 × 1 μm (Sydow 1897). van der Aa and Vanev (2002) transferred *P. allescheri* to *Asteromella allescheri*. The Chinese specimens had ellipsoidal conidia that measured 3–6 × 1.5–2 μm, without mucilaginous sheaths and apical appendages (Bai 2003, p. 218). The length to width ratio is different from *Asteromella allescheri*. The Chinese specimens may be a small-spored *Phoma* species.

7. *Phyllostcta amaranthi* Ellis & kellerman, J. Mycol., 1: 4, 1885.

→ *Phoma macrostoma* var. *macrostoma* Mont.

Hosts: *Amaranthus tetroflexus, Amaranthus viridis* (Amaranthaceae).

Distribution: Liaoning, Jilin.

Specimens: HMSAU 1092, HMSAU 1093, HMSAU 343.

Notes: Two *Phyllosticta* species, *P. amaranthi* and *P. atriplicis*, have been described on *Amaranthus.* They were reclassified as *Phoma macrostoma* var. *macrostoma* (conidia subglobose, ellipsoidal to oblong, mainly asptate, 8.5–14 × 2.5–4 μm) (Boerema et al. 2004) and *Ascochyta caulina* (conidiogenous cells 4–7 μm diam, conidia 1–3-septate, irregularly multiguttulate, 12–27 × 3.5–7.5 μm), respectively. The Chinese specimens had elliptical, cylindrical, 1-celled, hyaline conidia with 2 guttules that measured 10–14 × 3–4 μm, without mucilaginous sheaths and apical appendages (Bai 2003, p. 48), which is most likely *Phoma macrostoma* var. *macrostoma*.

8. *Phyllosticta amaryllidicola* van der Aa, Studies in Mycology, 5: 27, 1973.

Host: *Lycoris radiata* (Amaryllidaceae).

Distribution: Yunnan.

Specimens: HMSAU 2334.

Notes: *Phyllosticta amaryllidicola* was mistakenly spelt as *P. amaryllicola* by Bai (2003, p. 48). The description of the specimen from China (Bai 2003, p. 48) matched Aa’s description of the type specimen (van der Aa 1973).

9. *Phyllosticta amaryllidis* Bresadola, Hedwigia 35: 55, 1896.

→ *Phoma* sp.

Host: *Sansevieria trifasciata* (Asperagaceae).

Distribution: Yunnan.

Specimens: HMSAU 2349.

Notes: Three *Phyllosticta* species, *P. amaryllidis, P. cycadina* and *P. sansevieriae* were reported from *Sansevieria* spp. (Farr and Rossman 2012*).* These species have been reclassified as *Asteromella amaryllidis, Asterromella* sp., and *Phoma* sp. respectively (van der Aa and Vanev 2002). In the description of the specimen of *P. amaryllidis* from China (Bai 2003, p. 50), the shapes and sizes of conidiogenous cells and conidia are smaller than those of *Asteromella amaryllidis* (van der Aa and Vanev 2002). The conidial size of the Chinese specimen is 3.5–4 × 1–1.5 μm (Bai 2003, p. 50), and matches *P. sansevieriae*, which has been reclassified as a *Phoma* species (Batista 1952; van der Aa and Vanev 2002). Both *P. amaryllidis* and *P. sansevieriae* maybe small-spored *Phoma* species, because they lack a mucilaginous sheath and an apical appendage.

10. *Phyllosticta ambrosioidis* Thümen, Contr. Myc. Lusit., 592, 1878.

→ *Asteromella* sp. or *Phoma* sp.

Host: *Chenopodium album* (Atripliceae).

Distribution: Heilongjiang, Jilin, Tibet.

Specimens: HMSAU 57, HMSAU 1317, HMSAU 2666.

Notes: The Chinese specimens had conidiogenous cells that are flask-shaped, 1-celled, hyaline, and conidia that are ovoid, ellipsoidal, 1-celled, hyaline, 3–5 × 1–1.5 μm, without mucilaginous sheaths and apical appendages (Bai 2003, p. 71). This species may be a small-spored *Phoma* or *Asteromella* species.

11. *Phyllosticta anacardiacearum* van der Aa, Studies in Mycology, 5: 31, 1973.

→ *Phyllosticta capitalensis* Henn.

Host: *Elaeocarpus glabripetalus* (Elaeocarpaceae).

Distribution: Zhejiang.

Specimen: *Elaeocarpus glabripetalus*, Hangzhou, Zhejiang.

Notes: Lou et al. (2009) reported leaf blight of *Elaeocarpus glabripetalus* in Zhejiang, China. The pathogen was identified as *P. anacardiacearum*, based on morphology and ITS sequence data (EU821356–EU821361). The ITS sequences of this record EU821356 was identical to the ITS sequence (JF261465) of the epitye strain of *Phyllosticta capitalensis* (CPC18848) (Bai 2003; Glienke et al. 2011). The conidial size of the Chinese specimen (10–13 × 6–8 μm) agree with the description given by Glienke et al. 2011) for *P. capitalensis* (11–12 × 6–7 μm).

12. *Phyllosticta angelicae* Saccardo, Michelia, 2: 620, 1880.

→ *Asteromella* sp.

Host: *Angelica dahurica* (Apiaceae).

Distribution: Liaoning.

Specimen: HMSAU 1527.

Notes: The current name of *P. angelicae* is *Asteromella angelicae* (Sacc.) Moesz ex Bat. & Peres (Batista and Peres 1961). The morphology of the Chinese specimen (Bai 2003, p. 215) indicates an *Asteromella* species, i.e. branched conidiophores, conidiogenous cells ampulliform, hyaline, 3–6 × 1.5–2 μm, conidia ellipsoidal, round at both ends, 1-celled, hyaline, with one guttule, 3–5 × 1.5–2 μm.

13. *Phyllosticta annonae* Henn. Hedwiga, 41: 104, 1902.

→ *Phoma* sp.

Host: *Annona squamosa* (Annonaceae).

Distribution: Yunnan.

Specimen: HMSAU 2335.

Notes: This species has been reclassified in *Phoma* which produce ellipsoid or ovoid conidia measuring 5.7–8.1 × 2. 6–3.2 μm (van der Aa and Vanev 2002). The conidia of specimen from China were smaller than those of *Phyllosticta annonae* (3–4 × 1.5–2 μm vs. 5.7–8.1 × 2. 6–3.2 μm) (Bai 2003, p. 51). This indicates that it is a small-spored *Phoma* species.

14. *Phyllosticta apii* Halsted, Rep. New. Jers. Agric. St., 253, 1891.

→ *Phoma* sp.

Host: *Saposhnikovia divaricata* (Apiaceae).

Distribution: Liaoning.

Specimen: HMSAU 2350.

Notes: Bai (2003, p. 216) described specimens from China as conidiogenous cells ampulliform, 1-celled, hyaline, 5–8 × 2–3 μm, conidia oblong or long-fusiform, 1-celled, hyaline, 7–9 × 2–3.5 μm. As the conidia lacked a mucilaginous sheath and an apical appendage, this species most likely represents a *Phoma* species.

15. *Phyllosticta apocyni* Ellis & Martius, New North Am. Fungi in Am. Nat. Dec., p. 1264, 1884.

→ *Phyllosticta apocyni-androsaemifolii* Bubák & Dearness

Host: *Ecdysanthera rosea* (Apocynaceae).

Distribution: Yunnan.

Specimen: HMSAU 2340.

Notes: The specimens from China had conical conidiogenous cells, 3–6 × 2–4 μm; conidia globose or subglobose, 1-celled, 10–12 × 7–8 μm, with a mucilaginous sheath and an apical appendage, 5–13 μm long (Bai 2003, p. 53). This is in accordance with the description of *P. apocyni-androsaemifolii*, which is a synonym of *Phyllosticta apocyni* Ellis & Martius (van der Aa and Vanev 2002).

16. *Phyllosticta arecae* Höhnel, Sber. Akad. Wiss. Wien math. Naturw Kl., 1, 121; 385, 1912.

Host: *Areca catechu* (Areceae).

Distribution: Hainnan.

Specimen: HMSAU 2216.

Notes: The Chinese specimen (Bai 2003, p. 153) is typical *P. arecae* based on the similarity of the leaf spots (pale brown, with broad darker brown margin), pycnidia (globose or subglobose, 65–100 μm in diameter), conidiogenous cells (conical, 7.5–10 × 5–6 μm), and conidia (ovoidal, ellipsoidal or cylindrical, with guttules, 6–12.5 × 5–7 μm) with the original description (Höhnel 1912).

17. *Phyllosticta argyrea* Spegazzini, Fung. Arg. Pug., 2: 121. 1880.

→ *Phoma* sp.

Host: *Elaeagnus umbellata* (Elaeagnaceae).

Distribution: Henan, Liaoning, Shandong, Zhejiang.

Specimen: HMSAU 2000.

Notes: The conidia of Chinese specimen are longer than *Phyllosticta argyrea* (ovoid, acute at one or both ends, 1-celled, hyaline, 5–8 × 1.5–2 μm), and lack a mucilaginous sheath and an apical appendage (Bai 2003, p. 86), which indicates that it belongs to *Phoma*.

18. *Phyllosticta arida* Earle, Torr. Bot. Cl, 367, 1898.

→ *Phyllosticta minima* (Berkeley & M.A. Curtis) Underwood & Earle

Host: *Acer cinnamomifolium* (Aceraceae).

Distribution: Hainan, Jilin.

Specimens: HMSAU 2618.

Notes: *P. arida* is a synonym of *P. minima* (van der Aa and Vanev 2002). Based on the the conidial size and presence of a mucilaginous sheath, as well as host species, the Chinese specimen (Bai 2003, p. 47) is in accordance with *P. minima* (van der Aa 1973).

19. *Phyllosticta astragalicola* Massalongo, Bot. Centr., 26: 386, 1890.

→ *Asteromella* sp.

Host: *Astragalus tibetanus* (Fabaceae).

Distribution: Xinjiang.

Specimen: HMSAU 2223.

Notes: Two species of *Phyllosticta, P. astragali* and *P. astragalicola*, have been described from *Astragalus* spp. These two species have conidial sizes of 13–16 × 3 μm and 3–4 × 1–1.5 μm, respectively (Saccardo 1884, 1892), and each lack a mucilaginous sheath and an apical appendage, which thus do not belong to *Phyllosticta*. The Chinese specimen had conidia that are ellipsoidal or bacilliform, 4–6 × 1–1.5 μm (Bai 2003, p. 115), which is longer than the original description of *P. astragalicola*. The Chinese specimen is likely an *Asteromella* species.

20. *Phyllosticta atractyli* (Sicard) Koval, Jour. Bot. Acad. Sci. Ukr., 18(2): 75, 1961.

→ *Phoma* sp.

Host: *Atractylodes japonica* (Asteraceae).

Distribution: Liaoning.

Specimen: HMSAU 2336.

Notes: The conidia of the Chinese specimen (Bai 2003, p. 76) were longer than *Phyllosticta atractyli* (6–8 × 2–2.5 μm vs. 4–5 × 2–2.5 μm) (van der Aa and Vanev 2002) and also lacked a mucilaginous sheath and an apical appendage. It may represent a *Phoma* species.

21. *Phyllosticta bauhiniae* Cooke, Grevillea., 12: 26, 1883.

→ *Phoma* sp.

Host: *Bauhinia variegata* (Fabaceae).

Distribution: Yunnan.

Specimens: HMSAU 2338, HMSAU 2339.

Notes: The only known species described from *Bauhinia* spp. is *P. capitalensis. Phyllosticta bauhinicola* described from *Bauhinia* sp. was considered to represent *Phoma macrostoma* var. *macrostoma* (van der Aa and Vanev 2002), with conidia ovoidal or short cylindrical, 3–8.5 × 1.2–3.6 μm. The morphology of *P. bauhiniae* in the original description is similar to *P. bauhinicola* (conidia ellipsoidal, 7.5 × 2 μm) (Cooke 1883). The Chinese specimens had conidia size of 5–7 × 1.5–2 μm (Bai 2003, p. 116) and represent a *Phoma* species.

22. *Phyllosticta boussingaultiae* Spegazzini, Fg. Agr. novi v. Crit., 312, 1899.

→ *Phoma* sp.

Host: *Basella rubra* (Basellaceae).

Distribution: Yunan.

Specimen: HMSAU 2337.

Notes: The Chinese record is the only known report of *Phyllosticta* species from *Basella*. The morphological description (Bai 2003, p. 58) indicated that it is a *Phoma* species with small, ellipsoidal to ovoid conidia. Several *Phoma* species have been reported from *Basella* spp., including *Phoma albae* and *Phoma exigua*, the later has subsequently been reclassified as *Boeremia exigua* var. *exigua* (Aveskamp et al. 2010).

23. *Phyllosticta bejeirinckii* Vuillemin, Journ. de Bot., 2: 255, 1888.

→ *Phoma* sp.

Host: *Prunus mume* (Rosaceae).

Distribution: Guangdong.

Specimen: HMSAU 2218.

Notes: *Phyllosticta beijerinckii* has conidia that are ellipsoidal, hyaline, 6 × 5 μm. The conidia of the Chinese specimen (Bai 2003, p. 179) are longer (7.5–10 × 5–6 μm) than those of *P. beijerinckii*, and lack a mucilaginous sheath and an apical appendage. This may be a large-spored *Phoma* species.

24. *Phyllosticta berberidis* Rabenhorst, Herb. Myc. N., 1865, 1860.

→ *Phoma* sp.

Host: *Mahonia bealei* (Berberidaceae).

Distribution: Jilin.

Specimen: HMSAU 1168.

Notes: The conidia of *P. berberidis* reported from China are 1-celled, ellipsoidal or cylindrical, 5–6 × 2–3 μm and lack a mucilaginous sheath and an apical appendage (Bai 2003, p. 59). *Phyllosticta berberidis* has been reclassified as *Phoma macrostoma* var. *macrostoma* (van der Aa and Vanev 2002). However, the conidia of *Phoma macrostoma* var. *macrostoma* are 0–3–septate, variable in shape, 5–14 × 2.5–4 μm. The Chinese specimen represents another *Phoma* species with small conidia.

25. *Phyllosticta brassicae* (Currey) Westendorp, Bull. Brux. 397, 1851.

→ *Phoma lingam* (Tode) Desm.

Host: *Brassica oleracea* (Brassicaceae).

Distribution: Hebei, Jilin, Sichuan.

Specimen: HMSAU 702.

Notes: In the original description of *Phyllosticta brassicae* (Westendorp 1851), the conidial size was not given and this species has since been reclassified as *Phoma lingam* (van der Aa and Vanev 2002; Boerema et al. 2004). Boerema et al. (2004) considered *Phoma lingam* had ellipsoidal or sub-ellipsoidal conidia, with 2 polar guttules, and measured 3.5–4.5 × 1–1.5 μm. In Bai’s (2003, p. 82) Chinese record, the fungus was parasitic on leaves of *Brassica* spp., with ampulliform, 1-celled and hyaline conidiogenous cells, and ellipsoidal, hyaline, 1-celled, conidia that measured 3–5 × 1.5–2 μm. The characters are in accordance with *Phoma lingam*.

26. *Phyllosticta camelliae* Kickx, Flor. Crypt. Flandr, 1–490, 1867.

→ *Phoma* sp.

Host: *Camellia sinensis* (Theaceae).

Distribution: Anhui, Sichuan.

Specimens: HMSAU 2251, HMSAU 2633.

Notes: *Phyllosticta camelliae* has been synonymized with *Phomopsis theae* by van der Aa and Vanev (2002), which produces conidia ellipsoidal, fusiform, 1-celled, hyaline, with 2 guttules, 6–9 × 2–3 μm. The Chinese specimens had short conidia, (ellipsoidal, round at both ends, 1-celled, hyaline, with 2 guttules, 4–6 × 2–2.5 μm) and the short unbranched conidiogenous cells (ampulliform, 1-celled, hyaline 5–7.5 × 4–5 μm) (Bai 2003, p. 207). This indicates that this specimen belong to a *Phoma* species.

27. *Phyllosticta cannabis* (L.A. Kirchner) Spegazzini, Nov. Add. n. 150, 1875.

→ *Phoma cannabis* (L.A. Kirchn.) McPartl.

Host: *Cannabis sativa* (Cannabaceae).

Distribution: Jilin, Liaoning.

Specimens: HMSAU 707, HMSAU 1849, HMSAU 478.

Notes: *Phyllosticta cannabis* has been synonymized with *Phoma cannabis* (L.A. Kirchner) McPartland (1994). The host and morphology of the Chinese specimens (Bai 2003, p. 140) are in accordance with the original description (McPartland 1994).

28. *Phyllosticta capitalensis* Henn. Hedwigia, 48: 13, 1908.

Hosts: *Citrus* spp. (Rutaceae), *Annona squamosa* (Annonaceae), *Thea sinensis* (Theaceae), *Dracaena cambodiana* (Asparagaceae), *Aquilaria sinensis* (Thymelaeaceae), *Musa* sp. (Musaceae).

Distribution: Chongqing, Guizhou, Fujian, Zhejiang, Shandong, Yunnan.

Specimens: GZAAS 6.1201, GZAAS 6.1242, GZAAS 6.1202.

Notes: *P. capitalensis* is an endophyte with wide geographic distribution and range of hosts (Baayen et al. 2002; Glienke et al. 2011). Based on the ITS sequence of epitype designated by Glienke et al. (2011) and recent reports (Lou et al. 2009; Lin et al. 2010; Jin 2011; Xing et al. 2011; Wang et al. 2012; Wu et al. 2014), *P. capitalensis* is widely distributed in China.

29. *Phyllosticta caprifolii* (Opiz) Saccardo, Micheia, 1: 137, 1878.

→ *Phoma* sp.

Host: *Lonicera praeflorens* (Caprifoliaceae).

Distribution: Liaoning.

Specimen: HMSAU 1528.

Notes: Other than the record by Bai (2003, p. 64), species of *Phyllosticta* have not been recorded on *Lonicera* spp. The specimen from China produced 1-celled ovoid conidia that measured 4–6 × 2–3.5 μm. It is likely a *Phoma* species.

30. *Phyllosticta capsici* Spegazzini, Fg. Arg. Novi. V. Crit., 314, 1899.

→ *Phoma* sp.

Host: *Capsicum annuum* (Capsiceae).

Distribution: Jilin, Tibet.

Specimens: HMSAU 636, HMSAU 2765.

Notes: The conidia of specimen from China (Bai 2003, p. 204) are smaller (4–6 × 1.5–2.5 μm) than *Phyllosticta capsici* (7–8 × 3.5–4 μm) (Saccardo 1902). The Chinese specimens had conidia that lacked a mucilaginous sheath and an apical appendage, which indicates a *Phoma* species.

31. *Phyllosticta caraganae* Sydow, Hedwigia, 134, 1899.

→ *Phoma macrostoma* Mont.

Host: *Caragana microphylla* (Fabaceae).

Distribution: Inner Mongolia.

Specimen: HMSAU 2255.

Notes: *Phyllosticta caraganae* has long-oval conidia that measured 5–7 × 2–2.5 μm (Sydow 1899). van der Aa and Vanev (2002) considered that this species was *Phoma macrostoma*. The morphology of Chinese specimens (Bai 2003, p. 118) matches the original descriptions of *Phoma macrostoma* (Montagne 1849).

32. *Phyllosticta carochlae* N. Zhou et al. Fungal Biol. X: X, 2015.

Host: *Caryota ochlandra* (Palmae).

Distribution: Fujian.

Specimens: HMAS 245578, CGMCC 3.17317, CGMCC 3.17318.

Note: This is an accepted *Phyllosticta* species with typical morphology and well inferred phylogenetic relationships (Zhou et al. 2015).

33. *Phyllosticta castaneae* Ellis & Everhart, Proc. Acad. Phil., 2357, 1894.

→ *Phomopsis* sp.

Hosts: *Castanea mollissima* (Fagaceae), *Castanopsis sclerophylla* (Fagaceae), *Lithocarpus* sp. (Fagaceae).

Distribution: Anhui, Hainan, Jilin.

Specimens: HMSAU 1127, HMSAU 2659, HMSAU 2647.

Notes: The conidia of *P. castaneae* are hyaline, 6–8 × 2.5–3 μm (Ellis and Everhart 1894; Saccardo 1895). However, van der Aa and Vanev (2002) observed poorly developed *Phomopsis* species on the holotype. The morphological characters of the Chinese specimens also point to a *Phomopsis* species with conidiogenous cells ampulliform, 1-celled, hyaline, 5–12 × 4–6 μm, and conidia oval, cylindrical, fusiform, 1-celled, hyaline, 6–8 × 2.5–3 μm (Bai 2003, p. 97).

34. *Phyllosticta celticola* Bubák & Kabák, Ann. Myc., 5: 42, 1907.

→ *Phoma macrostoma* var. *macrostoma* Mont.

Host: *Celtis bungeana* (Cannabaceae).

Distribution: Liaoning.

Specimen: HMSAU 2037.

Notes: *Phyllosticta celtidicola* was erroneously spelt as *P. celticola* by Bai (2003, p. 211). *Phyllosticta celtidicola* has been considered a synonym of *Phoma macrostoma* var. *macrostoma* (Gruyter et al. 2002).

35. *Phyllosticta chaenomelesicola* L. Yu & J. K. Bai, Acta Mycol. Sin. 14(3): 192, 1995.

→ *Phoma* sp.

Host: *Chaenomeles speciosa* (Rosaceae).

Distribution: Liaoning.

Specimen: HMSAU 1981 (holotype).

Notes: According to the morphological description of the type material, this species has holoblastic, 1-celled, hyaline conidiogenous cells, and ovoid, cylindrical, 1-celled, hyaline conidia that measure 8–11 × 5–6 μm (Bai 2003, p. 171), which is more typical for a *Phoma* species.

36. *Phyllosticta chrysanthemi* Ellis & Dearness, Canad. Rec. Sc., 268, 1893.

→ *Phoma* sp.

Host: *Dendranthema morifolium* (Asteraceae).

Distribution: Guangdong.

Specimen: HMSAU 2204.

Notes: The pycnidia of the type material of *Phyllosticta chrysanthemi* contained brown ovoid to ellipsoidal conidia that measured 4–5 × 2.5–3 μm (Saccardo 1895; van der Aa and Vanev 2002). This could be a *Microsphaeropsis* species. The conidia of the specimen from China were hyaline, ellipsoidal, oval, 4–6 × 2–2.5 μm, without mucilaginous sheaths and apical appendages (Bai 2003, p. 77), which indicates a *Phoma* sp.

37. *Phyllosticta cirsii* Desmazières, Ann. S. N., 31, 1847.

→ *Phoma* sp.

Host: *Cirsium* sp. (Asteraceae).

Distribution: Liaoning.

Specimen: HMSAU 1992.

Notes: This is a *Phoma* species with 1-celled, hyaline, ovoid, ellipsoidal conidia that measured 3–5 × 2–2.5 μm (Bai 2003, p. 73).

38. *Phyllosticta citriasiana* Wulandari, Crous & Gruyter, Fungal Diversity, 34: 23, 2009.

Host: *Citrus maxima* (Rutaceae).

Distribution: Fujian, Guangdong.

Specimens: ZJUCC 200901, ZJUCC 200914.

Notes: Wang et al. (2012) investigated *Phyllosticta* species associated with *Citrus* spp. in China and identified three species, *P. citriasiana, P. citricarpa* and *P. citrichinaensis*. These three taxa are true *Phyllosticta* spp. with typical morphology and confirmed phylogenetic placement (ITS GenBank number: JN791637, JN791597, JN791644).

39. *Phyllosticta citricarpa* (McAlpine) van der Aa, Stud. Mycol., 5: 40, 1973.

Hosts: *Citrus reticulata, Citrus sinensis* (Rutaceae).

Distribution: Chongqing, Jiangxi, Zhejiang.

Specimens: ZJUCC 200968, ZJUCC 200946, ZJUCC 200928.

References: Wang et al. (2012). Fungal diversity, 52, pp. 209–224.

Note: See notes under *Phyllosticta citriasiana*.

40. *Phyllosticta citrichinaensis* X.H. Wang, K.D. Hyde & H.Y. Li, Fungal Diversity, 52: 209, 2012.

Host: *Citrus maxima* (Rutaceae).

Distribution: Guangdong.

Specimen: ZJU 201006 (holotype).

References: Wang et al. (2012). Fungal diversity, 52, pp. 209–224.

Note: See notes under *Phyllosticta citriasiana*.

41. *Phyllosticta clematidis* Ellis & Dearness, New Spec. Canad. Fung. In Canad. Rec. Sc., 268, 1893.

→ *Phoma clematidina* (Thüm.) Boerema

Host: *Clematis heraeleifolia* (Ranunculaceae).

Distribution: Sichuan, Hainan.

Specimens: HMSAU 2643, HMSAU 2708.

Notes: *P. clematidicola* Brunad, *P. clematidis* Brunad and *P. clematidi*s have been described from *Clematis* spp. All have been reclassified as *Phoma clematidina* (van der Aa and Vanev 2002). The description of the Chinese specimens (Bai 2003, p. 164) matches the description of *Phoma clematidina* (Saccardo 1895, 1899).

42. *Phyllosticta cocos* Cooke, *Grevillea* 8: 94, 1880.

→ *Phomopsis cocoina* (Cooke) Punith.

Host: *Rhapis excelsa* (Arecaceae).

Distribution: Jilin.

Specimen: HMSAU 2658.

Notes: Punithalingam (1975) examined the types of *Phoma cocoina* Cooke, *Phyllosticta cocos* and *Phomopsis cocoes* Petch, and combined them into *Phomopsis cocoina* (Cooke) Punith. In the original description, the conidia of *Phomopsis cocoina* are ellipsoidal and 8 μm long. The conidia of the specimen from China are fusiform or ellipsoidal, 6–7.5 × 2–2.5 μm (Bai 2003, p. 155), which is in general accordance with *Phomopsis cocoina*.

43. *Phyllosticta coffeicola* Speg., Revta. Fac. Agron. Univ. nac. La Plata, 2: 345, 1896.

→ *Phoma* sp.

Host: *Coffea ababica* (Rubiaceae).

Distribution: Hainan.

Specimen: HMSAU 2214.

Notes: The Chinese specimen (Bai 2003, p. 191) had conidiogenous cells ampulliform, 1-celled, hyaline, 3–5 × 3–4 μm, conidia ellipsoidal or oval, 1-celled, hyaline, 3–4 × 1.5–2 μm, without mucilaginous sheaths and apical appendages. This specimen may be a small-spored *Phoma* species.

44. *Phyllosticta commelinicola* Young, Mycologia, 7: 144, 1915.

→ *Phoma* sp.

Host: *Commelina communis* (Commelinaceae).

Distribution: Liaoning.

Specimens: HMSAU 456, HMSAU 1847, HMSAU 2068.

Notes: The conidial size of the specimen from China was similar to that in the original description of *P. commelinicola* (8–17 × 3–5 μm vs. 9.6–14.4 × 4.8–7.2 μm) (Young 1915). However, the conidial shape is cylindrical rather than ovoid in the original description. The description of the Chinese specimens (Bai 2003, p. 72) also indicated that the conidia were medianly constricted, which may represents a large-spored *Phoma* sp. because the conidia lack a mucilaginous sheath and an apical appendage.

45. *Phyllosticta commonsii* Ellis & Everhart, Jour. Myc., 146, 1889.

→ *Phoma* sp.

Hosts: *Paeonia lactiflora, Paeonia suffruticosa* (Paeoniaceae).

Distribution: Jilin.

Specimens: HMSAU 918, HMSAU 980.

Notes: Ellis and Everhart (1889) described the conidia of *P. commonsii* as oblong or ellipsoidal, smoky hyaline, 4–5 × 2–2.5 μm. Examination of type specimen showed two fungi present, i.e. *Microsphaeropsis*-like species with pigmented conidia, as well as *Phoma exigua* (van der Aa and Vanev 2002). The Chinese specimens had conidiogenous cells ampulliform, 1-celled, hyaline, 4–6 × 1.5–2.5 μm, and conidia measured 4–7.5 × 2–2.5 μm, which indicates a *Phoma* species (Bai 2003, p. 164).

46. *Phyllosticta convallaricola* L. Yu & J. K. Bai, Acta Mycologica Sinica, 14(3): 193, 1995.

→ *Phoma* sp.

Host: *Convallaria keisei* (Asparagaceae).

Distribution: Jilin.

Specimen: HMSAU 995.

Note: *Phyllosticta convallaricola* has been considered a small-spored *Phoma* sp. by van der Aa and Vanev (2002).

47. *Phyllosticta coriariicola* Spegazzini, Fungi Chilenses, 138, 1910.

→ *Asteromella* sp.

Host: *Coriaria sinica* (Coriariaceae).

Distribution: Sichuan.

Specimen: HMSAU 2728.

Note: The Chinese specimen had bacilliform, 1-celled conidia (Bai 2003, p. 125), indicating an *Asteromella* species.

48. *Phyllosticta coryli* Westendorp, Bull. Acad. Roy. Belg., 19: 9, 1851.

→ *Boeremia exigua* (Desm.) Aveskamp, Gruyter & Verkley.

Host: *Corylus heterophylla* (Betulaceae).

Distribution: Jilin.

Specimen: HMSAU 1129.

Notes: *Phyllosticta coryli* has been reclassified as *Boeremia exigua* (van der Aa and Vanev 2002). The Chinese specimen had hyaline, ellipsoidal, conidia that measured 4–6.5 × 2–3 μm (Bai 2003, p. 60), which are similar to *Boeremia exigua* (Westendorp 1851).

49. *Phyllosticta cotoneastri* Allescher, Hedwigia, 1: 158, 1897.

→ *Phoma* sp.

Host: *Cotoneaster multiflorus* (Rosaceae).

Distribution: Liaoning.

Specimen: HMSAU 2043.

Notes: *Phyllosticta cotoneastri* was reclassified as *Phoma asiatica* by van der Aa and Vanev (2002). The Chinese specimen (5–7 × 2.5–4 μm) had conidia that are shorter and broader than the type of *Phoma asiatica* (Bai 2003, p. 173). The Chinese specimen may be an unidentified *Phoma* sp.

50. *Phyllosticta crastophila* Saccardo, Michelia, 1: 153, 1878.

→ *Phoma herbarum* Cooke.

Hosts: *Setaria italica, Setaria viridis, Setaria* spp. (Paniceae).

Distribution: Inner Mongolia, Jilin.

Specimens: HMSAU 656, HMSAU 1400, HMSAU 1132, HMSAU 1399.

Notes: The conidia of *P. crastophila* are oblong, with obtuse ends, 2-guttulate, hyaline, and measured 5–6 × 2 μm (Saccardo 1878); the species has been reclassified as *Phoma herbarum* (van der Aa and Vanev 2002). Chinese specimens associated with different hosts have conidia of different sizes (Bai 2003). On *Setaria italica*, the conidia are ovoid, cylindrical, 4–6 × 1.5–2 μm, and on *Setaria viridis*, the conidia are 7–10 × 2.5–3.5 μm; both lacked a mucilaginous sheath and an apical appendage (Bai 2003). The former matches the description of *Phoma herbarum*, while the fungus on *Setaria viridis* may represent another *Phoma* sp.

51. *Phyllosticta crataegicola* Saccardo, Michelia, 1; 1878.

→ *Phoma* sp.

Host: *Crataegus pinnatifida* (Rosaceae).

Distribution: Jilin, Liaoning.

Specimens: HMSAU 892, HMSAU 1261, HMSAU 891, HMSAU 1057.

Notes: The Chinese specimens appear to be a small-spored *Phoma* species, with ovoid, ellipsoidal conidia that measure 4–5 × 2. 5–3 μm, without mucilaginous sheaths or apical appendages (Bai 2003, p. 174).

52. *Phyllosticta cruenta* (Fries) Kickx, Flor. Crypt. Flandr. 1: 412, 1849.

Host: *Polygonatum macropodium* (Asparagaceae).

Distribution: Inner Mongolia.

Specimen: HMSAU 1771.

Note: The specimen from China is typical of *P. cruenta* (van der Aa 1973; Bai 2003, p. 125).

53. *Phyllosticta cucurbitacearum* Saccardo, Michelia, 1: 145, 1878.

→ *Phoma* sp.

Host: *Cucurbita moshata* (Cucurbitaceae).

Distribution: Jilin.

Specimen: HMSAU 981.

Note: This is a *Phoma* sp. with 1-celled, hyaline, ellipsoidal or cylindrical conidia that measure 5–7 × 2–2.5 μm, without a mucilaginous sheath or apical appendage (Bai 2003, p. 83).

54. *Phyllosticta cycadina* Passerini, Atti Accad. Naz. Lincei, Rendiconti Adunanze Solenni, Ser 4: 66, 1888.

→ *Asteromella* sp.

Host: *Cycas revoluta* (Cycadaceae).

Distribution: Liaoning.

Specimen: HMSAU 2461.

Notes: The conidia of *P. cycadina* are hyaline, bacilliform, 2.5 × 0.5–0.7 μm (Saccardo 1892), and resemble an *Asteromella* sp. (van der Aa and Vanev 2002). Conidia from the Chinese specimen are larger than the type of *P. cycadina* (3–4 × 1–1.5 μm vs. 2.5 × 0.5–0.7 μm) (Passerini 1888; Bai 2003, p. 85). The Chinese specimen is likely an *Asteromella* sp.

55. *Phyllosticta cynanchi* Brunaud, Glan. Mycol., Ser 2: 6, 1892.

→ *Phoma* sp.

Host: *Cynanchum auriculatum* (Apocynaceae).

Distribution: Jiangsu.

Specimen: HMSAU 2639.

Note: The description points to a *Phoma* species with conidia oblong to ovoidal, 5–7 × 3.5–5 μm (Bai 2003, p. 57).

56. *Phyllosticta dahliaecola* Brunaud, Champ. Saint., 429, 1887.

→ *Boeremia exigua* (Desm.) Aveskamp, Gruyter & Verkley.

Host: *Dahlia variabilis* (Asteraceae).

Distribution: Jilin.

Specimen: HMSAU 1179.

Notes: This species was reclassified as *Phoma exigua* (van der Aa and Vanev 2002), which was subsequently renamed *Boeremia exigua* (Aveskamp et al. 2010). The morphology of this specimen from China (Bai 2003, p. 75) is in accordance with *Boeremia exigua.*

57. *Phyllosticta dalbergiicola* Sydow, Bull. Herb. Boiss., 82, 1901.

→ *Phomopsis nivea* (Syd. & P. Syd.) van der Aa.

Host: *Dalbergia* sp. (Fabaceae).

Distribution: Hainan.

Specimen: HMSAU 2727.

Notes: *Phyllosticta dalbergiicola* has been reclassified as *Phomopsis nivea* (van der Aa and Vanev 2002). The description of the record from China (Bai 2003, p. 119) indicates conidia oblong, 5–7 × 2–3 μm, with 2 guttules, in accordance with *Phomopsis*.

58. *Phyllosticta desmodiicola* Diedicke, Ann. Myc., 14: 178, 1916.

→ *Phoma* sp.

Host: *Desmodium styracifolium* (Fabaceae).

Distribution: Guangdong.

Specimen: HMSAU 2202.

Notes: According to van der Aa and Vanev (2002), the type specimen of *P. desmodiicola* contains several species including *Phoma* spp., *Microsphaeropsis* sp. and *Didymella* sp. The original description points to a *Phoma* species with conidia ellipsoidal or cylindrical, 5–7 × 2–3 μm (Sydow 1916). The specimen from China has similar conidial characters (Bai 2003, p. 121), indicating a *Phoma* species.

59. *Phyllosticta deutziae* Ellis & Everhart, Hourn. Myc., 146, 1889.

→ *Phoma* sp.

Host: *Deutzia prunifolia* (Hydrangeaceae).

Distribution: Liaoning.

Specimen: HMSAU 1988.

Notes: In Ellis and Everhart’s (1889) original description, this species has sub-ellipsoidal, fuscous conidia, 4–5 × 3 μm. van der Aa and Vanev (2002) revised this species as *Microsphaeropsis olivaceae,* while in the specimen from China, the conidia are ellipsoidal, 1-celled, hyaline, bi-guttulate, 3–6 × 2–3.5 μm (Bai 2003, p. 198), which points to a *Phoma* species.

60. *Phyllosticta deutziicola* Petr., Annls Mycol. 12(5): 471, 1941.

Host: *Deutzia scabra* (Hydrangeaceae).

Distribution: Hebei, Liaoning.

Specimens: HMSAU 2638, HMSAU 2058.

Notes: *P. deutziicola* was wrongly spelt as *P. deutzicola* by Bai (2003, p. 197). This species has conidia ellipsoidal, hyaline, 2–5 × 1–4 μm (Petrak 1914), and was considered to be *Phoma pomorum* (van der Aa and Vanev 2002). In Bai’s description, the conidia of this record are bacilliform, 1-celled, hyaline, 3–4 × 1–1.5 μm, which differs from the type of *Phoma pomorum*. More information is needed to determine its classification.

61. *Phyllosticta digitalis* Bellynck, West. Exs., 1053, 1855.

→ *Phoma* sp.

Host: *Rehmannia glutinosa* (Rehmanniaceae).

Distribution: Jilin.

Specimen: HMSAU 1105.

Notes: *P. digitalis* was first reported from *Digitalis lutea*, with conidia ovoid, 2-guttules, 7 × 2.5 μm (Saccardo 1884). The Chinese record lacks a mucilaginous sheath and an apical appendage (Bai 2003, p. 200), which indicates a *Phoma* species.

62. *Phyllosticta draconis* Berkeley, in Welw. F. Port. 5, 1853.

→ *Phomopsis* sp.

Host: *Dracaena angustifolia* (Asparagaceae).

Distribution: Yunnan.

Specimen: HMSAU 2609.

Notes: This name *Phyllosticta draconis* Berkeley reported in 1853 is invalid. *P. draconis* Berk. ex Cooke and *P. draconis* Berk. ex P. Karst were described later, with conidia 7 × 3 μm and 20 × 3 μm, respectively (Cooke 1885; Karsten 1896). The Chinese specimen had fusiform, biguttulate conidia, without a mucilatigous sheath or apical appendage (Bai 2003, p. 130), which is typical for alpha-conidia of *Phomopsis* species.

63. *Phyllosticta eriobotryae* Thümen, Contr. Fl. Mic. Lit., 215, 1877.

→ *Phoma* sp.

Host: *Eriobotrya japonica* (Rosaceae).

Distribution: Jilin.

Specimen: HMSAU 1130.

Note: This is a *Phoma* species with oval, ellipsoidal, 1-celled, hyaline conidia that measure 3.5–6 × 2.5–3 μm (Bai 2003, p. 175).

64. *Phyllosticta eucommiae* F. X. Chao & P. K. Chi, Fungus Diseasea on Cultivated Medicinal Plants of Guangdong Province, 139, 1994.

→ *Phoma* sp.

Host: *Eucommia ulmoides* (Eucommiaceae).

Distribution: Guangdong.

Specimen: HMSAU 2221.

Notes: This species was first reported in China; both the original description and non-original description did not mention the existence of a conidial appendage and mucilaginous sheath (Chi 1994; Bai 2003, p. 91). This is likely a *Phoma* species with conidia ellipsoidal, oval, 10–12.5 × 5–7 μm (van der Aa and Vanev 2002).

65. *Phyllosticta euonymella* Saccardo, Michelia, 1: 138, 1878.

→ *Asteromella* sp.

Host: *Euonymus alatus* (Celastraceae).

Distribution: Liaoning.

Specimen: HMSAU 1748.

Note: This is an *Asteromella* species with small conidia, hyaline, 1-celled, baciliform, 3–4 × 0.7–0.8 μm (Bai 2003, p. 70).

66. *Phyllosticta euonymi-japonici* L.L. Liu & G.Z. Lu Mycosystema, 26: 171, 2007.

Host: *Euonymus japonica* (Celastraceae).

Distribution: Liaoning.

Specimen: IBE 0000989 (holotype).

Notes: This is the only *Phyllosticta* species have been reported from *Euonymus* (Liu and Lu 2007). The morphology of this species is typical for *Phyllosticta*.

67. *Phyllosticta forsythiae* Saccardo, Fung. Ital. 87. 1877.

→ *Phoma* sp.

Host: *Forsythia suspensa* (Oleaceae).

Distribution: Liaoning.

Specimen: HMSAU 2235.

Notes: The Chinese specimen had ovoid or ellipsoidal conidia, 4–6 × 2–3 μm (Bai 2003, p. 141). According to van der Aa and Vanev (2002), all collections of this species belong to *Phoma exigua*, which has already been reported from *Forsythia* spp. The record from China is also a *Phoma* species.

68. *Phyllosticta fragaricola* Desmazières & Robinson, Plant Crypt. Fr., 3: 686, 1859.

→ *Phomopsis* sp.

Host: *Fragaria ananassa* (Rosaceae).

Distribution: Liaoning, Jilin, Tibet.

Specimen: HMSAU 163, HMSAU 1845, HMSAU 984, HMSAU 2720.

Notes: This is a *Phomopsis* species with cylindrical, ampulliform, 1-celled, hyaline, branched conidiogenous cells, and cylindrical, ellipsoidal, 1-celled, hyaline conidia that measured 4–7 × 1.5–2 μm (Bai 2003, p. 70).

69. *Phyllosticta gelsemii* Ellis & Everhart, Journ. Myc., 7: 131, 1892.

Host: *Strychnos nux-vomica* (Loganiaceae).

Distribution: Hainan.

Specimen: HMSAU 2180.

Notes: *P. gelsemii* was first described from *Gelsemium* sp. (Ellis and Everhart 1892). van der Aa transferred this species to *Colephoma* (van der Aa and Vanev 2002). The Chinese specimen (Bai 2003, p. 132) is different from *Colephoma gelsemii* in producing broader conidiogenous cells (7.5–10 × 5–7.5 μm vs. 4–12 × 2.5–5 μm) and shorter conidia (7.5–12.5 × 5–6 μm vs. 14–21 × 2.5–3 μm). More information is needed to confirm the identity of the Chinese specimen.

70. *Phyllosticta ghaesemnillae* Kooders, Botan, Untersuch., 205, 1907.

Host: *Codiaeum variegatum* (Euphorbiaceae).

Distribution: Yunnan.

Specimen: HMSAU 2341.

Notes: Though Bai (2003, p. 92) did not indicate the existence of conidial appendages, other morphological characters (pycnidia 105–135 μm in diameter; conidiogenous cells cylindrical or conical, 4–6 × 2–3.5 μm; conidia ovoidal, with one guttule and mucilaginous sheath, 10–13 × 7–7.5 μm) are in good accordance with the descriptions of Kooders and van der Aa and Vanev (2002). The Chinese specimen refers to a *Phyllosticta* species.

71. *Phyllosticta ginkgo* Brunaud, Liste Sphaerops., 7: 1886.

→ *Phoma* sp.

Host: *Ginkgo biloba* (Ginkgoaceae).

Distribution: Liaoning.

Specimen: HMSAU 1990.

Notes: The Chinese specimen had conidia that are ovoid, ellipsoidal, 4–5 × 2–2.5 μm, lacking a mucilaginous sheath and an apical appendage (Bai 2003, p. 99). This description points to a small spored *Phoma* species.

72. *Phyllosticta glycines* Thüm., Inst. Rev. Sci. Litt, Coimbra Ser. 3: 28:504, 1881.

→ *Phoma* sp.

Hosts: *Glycine* spp. (Fabaceae).

Distribution: Inner Mongolia, Jilin, Liaoning.

Specimens: HMSAU 432, HMSAU 573, HMSAU 993, HMSAU 926, HMSAU 1401, HMSAU 1531.

Notes: The Chinese specimens (Bai 2003, p. 121) had ampulliform, 1-celled, hyaline conidiogenous cells and oval, ellipsoidal, 1-celled, hyaline conidia that measured 4–7 × 2–3 μm, lacking a mucilaginous sheath and an apical appendage. This specimen belongs to a *Phoma* sp.

73. *Phyllosticta grossulariae* Saccardo, Michelia, 1: 136, 1878.

→ *Phoma* sp.

Host: *Ribes pauciflorum* (Grossulariaceae).

Distribution: Liaoning.

Specimen: HMSAU 2082.

Notes: According to Boerema et al. (2004), *Phoma macrostoma* var. *macrostoma* has many synonyms, including *Phyllosticta grossulariae*. The conidial size of this specimen from China (5–6 × 2–3 μm) is smaller than that of *Phoma macrostoma* var. *macrostoma*. This might be another *Phoma* species because of the lack of a mucilaginous sheath and an apical appendage (Bai 2003, p. 199).

74. *Phyllosticta guceviczii* Zhilina, Izv. Akad. Nauk. Armyan S. S. R., Biol. Sci., 15: 65, 1962.

→ *Asteromella* sp.

Host: *Dictamnus dasycarpus* (Rutaceae).

Distribution: Jilin.

Specimen: HMSAU 1852.

Notes: The conidia of this specimen are bacilliform, 1-celled, hyaline, 3–4 × 1–1.5 μm (Bai 2003, p. 192). This is an *Asteromella* species.

75. *Phyllosticta haynaldii* Roumeguére & Saccardo, Sacc., Michelia, 2: 342, 1880.

→ *Phoma* sp.

Host: *Ilex cornuta* (Aquifoliaceae).

Distribution: Liaoning.

Specimens: HMSAU 2342, HMSAU 2364.

Notes: The description of this specimen (Bai 2003, p. 17) showed that this record is a *Phoma* species with hyaline ovoidal, ellipsoidal conidia that measured 4–6.5 × 2–2.5 μm.

76. *Phyllosticta hemerocallidis* G. M. Chang & P. K. Chi, Chi et al., Fungus Diseases on Cultivated Medicinal Plants of Guangdong Province, 214, 1994.

→ *Phyllosticta hemerocallidis* (G. M. Chang & P. K. Chi) Vanev.

Host: *Hemerocallis fliva* (Xanthorrhoeaceae).

Distribution: Guangdong.

Specimen: HMSAU 2222.

Notes: *Phyllosticta hemerocallidis* (G. M. Chang & P. K. Chi) Vanev was first described as *Phyllostictina hemerocallidis* on cultivated medicinal plants (Chi 1994). It was transferred to *Phyllosticta* by van der Aa and Vanev (2002). In *Flora Fungorum Sinicorum* (Bai 2003, p. 128), this species was recorded with the wrong author name.

77. *Phyllosticta heveae* Zimmermann, Bull. Inst. Buttenuitenz., 10: 21, 1901.

→ *Phoma* sp.

Host: *Hevea* sp. (Euphorbiaceae).

Distribution: Jilin.

Specimen: HMSAU 2069.

Notes: van der Aa and Vanev reclassified this species as *Phomopsis ramicola* (van der Aa and Vanev 2002), while the sketch and description of specimen from China (Bai 2003, p. 94) indicates a *Phoma* species with conidia ellipsoidal or ovoid with obtuse or acute at both ends, 3–7 × 2–3 μm, without mucilaginous sheaths and apical appendages.

78. *Phyllosticta hostae* Y.Y. Su & L. Cai, Persoonia, 28: 76, 2012.

Host: *Hosta plantaginea* (Asparagaceae).

Distribution: Beijing.

Specimen: HMAS242924 (holotype).

Note: This is an accepted *Phyllosticta* species with typical morphology and well inferred phylogenetic relationships (Su and Cai 2012).

79. *Phyllosticta hubeiensis* K. Zhang & L. Cai, Mycol. Prog., 2012.

Host: *Viburnum odoratissimum* (Adoxaceae).

Distribution: Hubei.

Specimen: HMAS 243495 (holotype).

Note: This species is an accepted *Phyllosticta* species with typical morphology and well inferred phylogenetic relationships (Zhang, Zhang, et al. 2013).

80. *Phyllosticta iridis* Ellis & Everhart, New Fung. in Proceed. Acad. N. Sc. Philad, 456, 1893.

→ *Phoma* sp.

Host: *Iris ensata* (Iridaceae).

Distribution: Ningxia.

Specimen: HMSAU 2256.

Notes: According to the description of specimen from China (Bai 2003, p. 106), the conidia (ellipsoidal, oval or fusiform, 6–7.5 × 4–5 μm, without mucilaginous sheaths and apical appendages) are different from *Phyllosticta* species described form *Iris* spp., such as *P. iridis* (9–11 × 2.5 μm) *P. iridum* (10 × 2.5 μm) and *P. pseudacori* (5 × 2.5 μm). The description points to a *Phoma* species.

81. *Phyllosticta jasmini* Saccardo, Michelia, 1: 138, 1878.

Hosts: *Jasminum* spp. (Oleaceae).

Distribution: Tibet, Yunnan.

Specimens: HMSAU 2379, HMSAU 2343.

Notes: The description of the record in China indicates sheath surrounding the conidia, but no apical appendages were observed (Bai 2003, p. 143). Thus, there is not sufficient evidence to include this species in *Phyllosticta*. According to van der Aa and Vanev (2002), a second species, *Phoma* sp. has been observed from the holotype of *P. jasmini*. More information of the record from China is needed to confirm its identity.

82. *Phyllosticta jasminicola* (Desmazières) Saccardo, Syll. Fung., 11: 474, 1895.

→ *Phoma* sp.

Host: *Jasminum sambac* (Oleaceae).

Distribution: Yunan.

Specimen: HMSAU 2344.

Notes: Based on the type specimen of *P. jasminicola*, this species has been revised as *Asteromella jasminicola* by Petrak (1934). The conidial size of *Asteromella jasminicola* is 2–3 × 0.5 μm. In the description of the Chinese specimen (Bai 2003, p. 144), the conidia are ellipsoidal, 3–4 × 2–2.5 μm, without mucilaginous sheaths and apical appendages, which are larger than *Asteromella jasminicola*. According to the illustration and description, this may be a *Phoma* species.

83. *Phyllosticta jatrophae-podagricae* Yadav & Rao, J. Univ. Poona, 54: 155, 1981.

→ *Phomopsis* sp.

Host: *Jatropha podagrica* (Euphorbiaceae).

Distribution: Yunan.

Specimen: HMSAU 2345.

Notes: *Phyllosticta jatrophae-podagricae* is the only species associated with *Jatropha* spp. The type of *Phyllosticta jatrophae-podagricae* was regarded as a *Phoma* species (van der Aa and Vanev 2002). The specimen from China has conidia that are fusiform, acute ends, 6–8 × 2–2.5 μm (larger than the type), with 1–2 guttules (Bai 2003, p. 96). This is likely a *Phomopsis* species.

84. *Phyllosticta juglandis* Saccardo, Michelia, 1: 135, 1878.

→ *Phoma* sp.

Host: *Juglans sieboldana* var. *cordiformis* (Juglandaceae).

Distribution: Liaoning.

Specimen: HMSAU 2050.

Notes: The conidiogenous cells of this species are ampulliform, 1-celled, hyaline, 4–6 × 2–3 μm, and the conidia are ellipsoidal or cylindrical, 4–7 × 2.5–3 μm, without mucilaginous sheaths and apical appendages (Bai 2003, p. 107). The description also indicates that some of the conidia are brown. This record may be a *Phoma* species.

85. *Phyllosticta kalopanacis* G. Z. Lu & J. K. Bai, in Yu et al., Journal of Shenyang Agricultural University, 25(2): 156, 1994.

→ *Phoma* sp.

Host: *Kalopanax septemlobus* (Araliaceae).

Distribution: Liaoning.

Specimen: HMSAU 2047.

Notes: This name was first published without any description (Yu et al. 1994). The description of the Chinese specimen indicated that the conidia are fusiform, or ellipsoidal, pointed at both ends, 6–7.5 × 1.5–2 μm, without mucilaginous sheaths and apical appendages (Bai 2003, p. 55). This species may be a *Phoma* species with small conidia.

86. *Phyllosticta lantanae* Passerini, Erb. Critt. It., 2: 1290, 1882.

→ *Phoma* sp.

Hosts: *Viburnum fordiae, Viburnum sargentii* (Adoxaceae).

Distribution: Liaoning, Taiwan.

Specimens: HMSAU 2635, HMSAU 2048.

Notes: The only accepted *Phyllosticta* species associated with *Viburnum* is *Phyllosticta hubeiensis* (Farr and Rossman 2012; Zhang, Su, et al. 2013), with conidia bearing mucilaginous sheaths and apical appendages. This record represents a *Phoma* species with conidia 1-celled, oval, 5–7.5 × 2–2.5 μm (Bai 2003, p. 65).

87. *Phyllosticta ligustri* Saccardo, Michelia, 1:134, 1878.

→ *Phoma* sp.

Host: *Ligustrum japonicum* (Oleaceae).

Distribution: Jilin.

Specimen: HMSAU 1058.

Notes: Fungus described by Saccardo was not observed from the type specimen of *P. ligustri* (van der Aa and Vanev 2002). In Saccardo’s original description, the conidia are ovoid, 6–7 × 2.5–3 μm, while the conidia of the specimen from China are shorter (3–5 × 2–3 μm), ellipsoidal, without mucilaginous sheaths and apical appendages (Bai 2003, p. 145). This may be a *Phoma* species.

88. *Phyllosticta lilii* Ellis & Dearness, in Canad. Rec. Scienc., 267, 1893.

→ *Phoma* sp.

Hosts: *Lilium brownie, Lilium souliei* (Liliaceae).

Distribution: Guangdong.

Specimens: HMSAU 2182, HMSAU 2161.

Notes: Ellis and Everhart (1893) described *P. lilii* as the pycnidial stage of *Leptosphaeria lilii*, with conidia 4–5 × 2.5–3 μm. van der Aa and Vanev (2002) regarded this species as typical for *Coniothyrium* with typical brownish conidia. However, the record from China has hyaline, globose or ellipsoidal conidia, 3–5 × 2–2.5 μm, without mucilaginous sheaths and apical appendages (Bai 2003, p. 131). This may be a *Phoma* species.

89. *Phyllosticta lindericola* Ellis & Everhart, Proc. Acad. Phil., 354, 1894.

→ *Phoma* sp.

Host: *Lindera* sp. (Lauraceae).

Distribution: Anhui.

Specimen: HMSAU 2700.

Notes: The conidia of *P. lindericola* are oblong or ellipsoidal, 4–7 × 2–3 μm (Ellis and Everhart 1894), while the conidia of the specimen from China are subglobose or ellipsoidal, 5–9 × 3–5 μm, without mucilaginous sheaths and apical appendages. Based on the type specimen, this could be a *Phomopsis* sp. (van der Aa and Vanev 2002), but the record from China is likely a *Phoma* species because of its ampulliform, 1-celled, hyaline conidiogenous cells, 5–7.5 × 5–6 μm (Bai 2003, p. 110).

90. *Phyllosticta linocierae* Thüm, Rev, Mycol. Toulouse 2:36, 1880.

→ *Phoma* sp.

Host: *Olea yunnanensis* (Oleaceae).

Distribution: Yunnan.

Specimen: HMSAU 2509.

Notes: The Chinese specimen lacks a mucilaginous sheath and an apical appendage (Bai 2003, p. 147). Its morphological description indicates a *Phoma* species.

91. *Phyllosticta liquidambaris-formosanae* J. K. Bai & G. Z. Lu. *Flora Fungorum Sinicorm*, 15: 104, 2002.

→ *Phoma* sp.

Host: *Liquidambar formosana* (Altingiaceae).

Distribution: Guangxi.

Specimen: HMSAU 2703.

Notes: In the original description, the conidiogenous cells of *P. liquidambaris-formosanae* are ampulliform, 1-celled, hyaline, 2–5 × 1.5–2 μm; the conidia are fusiform or oblong, 1-celled, hyaline, 5–7 × 2–2.5 μm, without mucilaginous sheaths and apical appendages (Bai 2003, p. 104). These morphological characters do not fit the the generic concept of *Phyllosticta* but indicate a *Phoma* species.

92. *Phyllosticta lonicerae* Westend., Bull. Acad. Brux, 18(2): 399, 1851.

→ *Phoma* sp.

Host: *Lonicera confusa* (Caprifoliaceae).

Distribution: Guangdong.

Specimen: HMSAU 2213.

Notes: *Phyllosticta lonicerae* has been treated as a synonym of *Kabatia periclymeni* (Sutton 1980; van der Aa and Vanev 2002). The description of the conidiomata of the specimen from China (Bai 2003, p. 147) is atypical for *Kabatia* (Saccardo 1906). The morphology of this record (conidiogenous cells ampulliform, 1-celled, hyaline, 7.5–10 × 4–6 μm, conidia cylindrical, ellipsoidal, 1-celled, hyaline, with 2 guttules, 6–9 × 2.5–3 μm, without mucilaginous sheaths and apical appendages) indicates a *Phoma* species.

93. *Phyllosticta ludwigiae* Peck, Ann. Rep. Reg. N. Y. St. Mus. 44:135, 1891.

→ *Phoma* sp.

Host: *Oenothera biennis* (Onagraceae).

Distribution: Sichuan.

Specimen: HMSAU 2624.

Notes: The conidia from the Chinese specimen are larger than the original description of *P. ludwigiae* (5–10 × 4–6 μm vs. 7–9 × 4 μm) and lack mucilaginous sheaths and apical appendages (Bai 2003, p. 152). No accepted *Phyllosticta* sp. has been reported from *Oenothera* spp. The description of the Chinese specimen indicates a *Phoma* species.

94. *Phyllosticta lychnidis* Bondartsev, Bull. Jard. Jard. Imper. Bot. St. Petersh., 12: 102, 1912.

Host: *Lychnis coronat*a (Caryophyllaceae).

Distribution: Liaoning.

Specimen: HMSAU 2083.

Notes: *Phyllosticta lychnidis* Bondartsev is a later homonym of *P. lychnidis* (Kuńze & J. C. Schmidt: Fries) Ellis & Everhart. Both names have been reclassified as *Boeremia exigua* (*Phoma exigua*) (van der Aa et al. 2000; van der Aa and Vanev 2002; Aveskamp et al. 2010). The morphology of specimen from China is generally similar to *Boeremia exigua*, but Bai (2003, p. 68) also mentioned the existence of short colloidal appendages; thus, more information is needed to confirm its identity.

95. *Phyllosticta lythri* Cejp, Nova Hedwigia, 18(2–4): 564, 1969.

→ *Boeremia exigua* (Desm.) Aveskamp, Gruyter & Verkley.

Host: *Lythrum salicaria* (Lythraceae).

Distribution: Jilin.

Specimen: HMSAU 2394.

Notes: Boerema and Dorenbosch (1973) reported *Phoma exigua* from *Lythrum* sp., which has been reclassified as *Boeremia exigua* (Aveskamp et al. 2010). The morphology of the Chinese specimen is in good accordance with the original description of *Boeremia exigua*, with subglobose or ellipsoidal or subglobose conidia that measure 4–6 × 2–2.5 μm (Bai 2003, p. 133).

96. *Phyllosticta macleayae* Naito, Memoirs of the College of Agriculture, Kyoto, 47: 46, 1940.

→ *Phoma* sp.

Host: *Macleaya cordata* (Papaveraceae).

Distribution: Jilin.

Specimen: HMSAU 1059.

Notes: The original description of *Phyllosticta macleayae* indicates the existence of septa in conidia (Naito 1940). van der Aa and Vanev (2002) pointed out that this species may be conspecific to *Phoma glaucii*. In the description of the Chinese specimen, Bai (2003, p. 156) indicated the existence of constrictions in the middle of the conidia, but no septum was observed. Based on morphology, this is a *Phoma* species.

97. *Phyllosticta magnoliae* Saccardo, Michelia, 1: 139, 1878.

Hosts: *Michelia alba, Magnolia denudata, Magnolia liliflora* (Magnoliaceae).

Distribution: Jilin, Liaoning, Sichuan.

Specimens: HMSAU 2080, HMSAU 2707, HMSAU 1060.

Notes: Various *Phyllosticta* records have been reported from *Michelia* and *Magnolia* species, including *P. magnoliae* Sacc., *P. cookie* Sacc., *P. magnolia-pumilae* Sawada, *P. yugokwa* Sawada and *P. kobus* P. Henn. All these species have been placed in *Phoma*. (van der Aa and Vanev 2002), except *P. kobus* which has typical conidia of *Phyllosticta*, 9–12 × 6–8 μm, with mucilaginous sheaths and apical appendages (Hennings 1905). The conidia of the specimen from China are ellipsoidal, 5–7 × 2.5–3 μm, lacking apical appendages (Bai 2003, p. 134), which do not place it in *Phyllosticta*.

98. *Phyllosticta malkoffii* Bubak, Ann. Myc., 6: 24, 1908.

→ *Boeremia exigua* (Desm.) Aveskamp, Gruyter & Verkley.

Host: *Gossypium herbaceum* (Malvaceae).

Distribution: Liaoning.

Specimen: HMSAU 6022.

Notes: This species has been synonymized with *Boeremia exigua* (Aveskamp et al. 2010). The Chinese specimen matches the original description of *Boeremia exigua* (Bai 2003, p. 136).

99. *Phyllosticta medicaginis* (Fuckel) Saccardo, Syll. Fung. 3: 42, 1884.

→ *Boeremia exigua* (Desm.) Aveskamp, Gruyter & Verkley.

Hosts: *Medicago sativa* (Fabaceae), *Trigonella foenum-graecum* (Fabaceae).

Distribution: Jilin, Xinjiang.

Specimens: HMSAU 512, HMSAU 2692.

Notes: *Phyllosticta bonanseana* and *P. medicaginis* have been described on *Medicago* sp. *P. trilobachne* and *P. medicaginis* have been described on *Trigonella* sp.; but none of these is an accepted species in *Phyllosticta. P. bonanseana, P. medicaginis, P. trilobachne* have been reclassified as *Phoma exigua* var. *exigua, Sporonema phacidioides* and *Phoma* sp., respectively (van der Aa and Vanev 2002). The current name of *Phoma exigua* var. *exigua* is *Boeremia exigua* (Aveskamp et al. 2010), with conidia variable in shape, hyaline, thin-walled, smooth, mainly aseptate, 2.5–12 × 2–4 μm. The conidia of specimens from China are cylindrical, 1-celled, hyaline, 5–9 × 1.5–2.5 μm, without appendages (Bai 2003, p. 122). Considering the morphology, the specimen from China belongs to *Boeremia exigua*.

100. *Phyllosticta menthae* Bresadola, Ann. Mycol., 13: 104, 1915.

→ *Boeremia exigua* var. *exigua* (Desm.) Aveskamp, Gruyter & Verkley.

Host: *Scutellaria baicalensis* (Lamiaceae).

Distribution: Liaoning.

Specimen: HMSAU 2451.

Notes: *P. decidua* (conidia cylindrical, 3–3.5 × 1.5 μm) (Ellis and Kellerman 1883), *P. menthae* (conidia ellipsoidal or cylindrical, 6–7 × 2–2.5 μm) (Bresadola 1915), and *P. scutellariae* (oblong or ellipsoidal, 5–6 × 2.5–3 μm) (van der Aa and Vanev 2002) have been described from *Scutellaria* spp. All these three species have been reclassified as *Boeremia exigua* var. *exigua* (Aveskamp et al. 2010). The morphology of the specimen from China (conidiogenous cells ampuliform, 5–6 × 3–4 μm, conidia ellipsoidal or cylindrical, 4–7 × 2.5–3 μm, without mucilaginous sheaths and apical appendages) (Bai 2003, p. 108) are essentially similar to *Boeremia exigua* var. *exigua*.

101. *Phyllosticta musaechinensis* S.P. Wu, Z.Y. Liu & K.D. Hyde, Phytotaxa, 188: 142, 2014.

Host: *Musa* sp.

Distribution: Chongqing.

Specimens: GZAAS6.1247, GZAAS6.1384.

Note: This species is an accepted *Phyllosticta* species with typical morphology and strong phylogenetic inference (Wu et al. 2014; ITS GenBank number: KF955294).

102. *Phyllosticta murrayicola* van der Aa, Studies in Mycology, 5: 71, 1973.

Host: *Murraya paniculata* (Rutaceae).

Distribution: Yunnan.

Specimen: HMSAU 2346.

Notes: The description of leaf spots (ochreous, with purple brownish border), pycnidia (globose or subglobose, 80–175 μm in diameter) and conidia (ovoidal, 10–12.5 × 6–8 μm) of the Chinese specimen (Bai 2003, p. 192) is in accordance with the description of type material of *Phyllosticta murrayicola* (van der Aa 1973).

103. *Phyllosticta musarum* (Cooke) van der Aa, Studies in Mycology, 5: 72, 1973.

Host: *Musa* spp. (Musaceae).

Distribution: Hainan.

Specimen: Not provided in literature.

Notes: Pu et al. (2008) isolated *Phyllosticta* species from *Musa* in China, and morphologically identified as *P. musarum* with conidia ‘1-celled, obovoidal, ellipsoidal or short cylindrical, pyriform when young, with a truncate base, broadly rounded, 15–18 × 9–10 µm’. The microscopic photographs clearly showed conidia with mucilaginous sheaths and apical appendages, typical of *Phyllosticta*. More recent molecular studies reveal three *Phyllosticta* species causing freckle diseases on *Musa* (Glienke et al. 2011; Wang et al. 2012, Wikee et al. 2013); DNA sequence analysis is needed to determine if the Chinese specimens belong to *Phyllosticta musarum*.

104. *Phyllosticta nigro-maculans* Saccardo, Ann. Mycol. 13: 134, 1915.

→ *Asteromella* sp.

Hosts: *Pulsatilla chinesis* (Ranunculaceae), *Anemone rivularis* (Ranunculaceae).

Distribution: Hebei, Liaoning.

Specimens: HMSAU 2033, HMSAU 66338.

Notes: There are two *P. nigro-maculans* records, *P. nigro-maculans* Sacc. 1896 from *Orchis epiphytarum* and *P. nigro-maculans* Sacc. 1915 from *Anemone nemorosa*. Both have small, hyaline, cylindrical conidia, 5–6 × 1 μm and 3–3.6 × 1 μm (Saccardo 1896, 1915). The Chinese specimens had conidia ellipsoidal, hyaline, 2–3 × 1–1.5 μm, without mucilaginous sheaths and apical appendages (Bai 2003, p. 166), similar to an *Asteromella* species.

105. *Phyllosticta nitidula* Durieu & Mont. Syll. Gen. Sp. Crypt. (Paris): 279, 1856.

→ *Asteromella* sp.

Host: *Lonicera japonica* (Caprifoliaceae).

Distribution: Jilin.

Specimen: HMSAU 1420.

Notes: *Phyllosticta alpigena* has been reported from *Lonicera nigra* (Saccardo 1903); its conidia are 4–4.5 × 1 μm. This species has been transferred to *Asteromella* as *A. alpigena*. The Chinese record (Bai 2003, p. 64) should not be classified as *Phyllosticta* because of its small conidia (2–4 × 1 μm), and absence of mucilaginous sheaths and apical appendages. It is likely an *Asteromella* species.

106. *Phyllosticta osmanthi* Tassi, Bull. Labor. Ort. Bot. Siena, 142, 1899.

→ *Phoma* sp.

Host: *Osmanthus fragrans* (Oleaceae).

Distribution: Liaoning.

Specimen: HMSAU 2347.

Notes: The description (Bai 2003, p. 146) indicates a *Phoma* species, with conidia ellipsoidal, obtuse at both ends, 4–5 × 1.5–2 μm, without mucilaginous sheaths and apical appendages.

107. *Phyllosticta osmanthicola* Trichieri, Bull. Orto Bot. Napoli, 3: 4, 1911.

→ *Phoma* sp.

Host: *Osmanthus fragrans* (Oleaceae).

Distribution: Taiwan.

Specimen: HMSAU 2637.

Notes: van der Aa revised this species as *Phomopsis osmanthi* based on the original description (Saccardo and Trotter 1931; van der Aa and Vanev 2002). The morphology of Chinese specimen is somewhat similar with the original description (long-ellipsoidal, 1-celled, hyaline, with 2 guttules, 6–8.5 × 2–2.5 μm vs. fusoid, 1-celled, hyaline, with 1–2 guttules, 7–9.5 × 2 μm without mucilaginous sheaths and apical appendages) (Bai 2003, p. 147), while the ampulliform, 1-celled, hyaline conidiogenous cells showed this record should be a *Phoma* species.

108. *Phyllosticta osteospora* Saccardo, Michelia, 1: 531, 1878.

Host: *Fraxinus rhynchophylla* (Oleaceae).

Distribution: Liaoning.

Specimen: HMSAU1982.

Notes: This species was reclassified as *Asteromella osteospora* by Rupprecht (1959), with average conidial size of 7.2 × 1 μm. According to the description and illustration of specimen from China, the conidia are dumb-bell-shaped, 3–6 × 0.7–1 μm, which is shorter than the type of *Asteromella osteospora* (Bai 2003, p. 149). This record is more likely the spermatial stage of a *Phyllosticta* species. Currently, there is no accepted species reported from *Fraxinus* spp.; therefore, more information is needed for this Chinese record.

109. *Phyllosticta panax* Nakata & Takimoto, in Hara, Pathologia Agriculturalis Plantarum, 5, 1921.

→ *Phoma* sp.

Host: *Panax ginseng* (Araliaceae).

Distribution: Jilin.

Specimen: HMSAU 988.

Notes: Bai’s (2003, p. 55) identification of this species was based on Chi et al. (1966) and the host. The description indicated a species with conidiogenous cells ampulliform, hyaline, 6–7.5 × 4–5 μm, conidia ovoid, ellipsoidal, 1-celled, hyaline, 4–6 × 1.5–2 μm, without mucilaginous sheaths and apical appendages. This points to a small-spored *Phoma* species.

110. *Phyllosticta papaya* Saccardo, Malpighia 5: 274, 1891.

→ *Asteromella* sp.

Host: *Carica papaya* (Caricaceae).

Distribution: Guangdong.

Specimen: HMSAU 2461.

Notes: Both *Phyllosticta papaya* Saccardo and *P. caricae-papayae* Allesch. have been reported from *Carica papaya* (Saccardo 1891; Hennings 1895). The descriptions of these two species indicate they are *Asteromella* spp. The conidia of the specimen from China were larger than *P. papaya and P. caricae-papayae* (4–5 × 1.5–2 μm, without mucilaginous sheaths and apical appendages) (Bai 2003, p. 67). This is an *Asteromella* species.

111. *Phyllosticta pharbitis* Saccardo, Michelia, 1: 144, 1878.

→ *Phoma* sp.

Host: *Pharbitis hederacea* (Convolvulaceae).

Distribution: Jilin.

Specimen: HMSAU 985.

Notes: The original description of this species indicates a *Phoma* sp. with conidia oblong, 6 × 2–3 μm (Saccardo 1878). The description of the specimen from China is in good accordance with the original description, with conidia 3–6 × 2–2.5 μm, without mucilaginous sheaths and apical appendages (Bai 2003, p. 79).

112. *Phyllosticta phaseolina* Saccardo, Michelia, 1: 149, 1878.

→ *Boeremia exigua* (Desm.) Aveskamp, Gruyter & Verkley.

Hosts: *Dolichos lablab* (Fabaceae), *Phaseolus calcaratus, P. vulgaris* (Fabaceae), *Vigna sinensis* (Fabaceae).

Distribution: Inner Mongolia, Jilin, Liaoning.

Specimens: HMSAU 983, HMSAU 852, HMSAU 989, HMSAU 324, HMSAU 2006, HMSAU 703, HMSAU 851.

Notes: In the original description of *P. phaseolina*, the conidia are oval or ellipsoidal, 6 × 2.5 μm. van der Aa and Vanev (2002) reclassified this species as *Phoma exigua*, which was treated as a synonym of *Boeremia exigua* (Aveskamp et al. 2010). The conidia of the specimen from China are ovoid or ellipsoidal, 3–6 × 2–2.5 μm (Bai 2003, p. 111); the morphology is in good accordance with *Boeremia exigua*.

113. *Phyllosticta phellodendri* Negru, in Stud. Univ. Victor babes et Bolyaik Romania, 3: 14, 1958.

→ *Phoma* sp.

Host: *Phellodendron amurense* (Rutaceae).

Distribution: Jilin.

Specimen: HMSAU 1267.

Notes: Based on the original description, van der Aa (1973) indicates this species may be a *Phomopsis* or large-spored *Phoma* species with conidia ellipsoidal, hyaline, 1-celled, 8–10 × 3–3.5 μm (van der Aa and Vanev 2002). The Chinese specimen had smaller conidia lacking mucilaginous sheaths and apical appendages (Bai 2003, p. 194), which points to a small-spored *Phoma* species.

114. *Phyllosticta phlogis* Vestergen, Cefv. K. Vet-Akad. Forh. Jan., 1: 37, 1897.

→ *Phoma* sp.

Host: *Phlox paniculata* (Polemoniaceae).

Distribution: Liaoning.

Specimen: HMSAU 2054.

Notes: The Chinese specimen (Bai 2003, p. 157) had conidia that are 1-celled, hyaline, oval, ellipsoidal, 3–5 × 2–2.5 μm, without mucilaginous sheaths and apical appendages. This is a *Phoma* species.

115. *Phyllsoticta photinica* Saccardo, Michelia, 1: 276, 1878.

→ *Asteromella* sp.

Host: *Photinia glabra* (Rosaceae).

Distribution: Guizhou.

Specimen: HMSAU 2726.

Notes: No fungus described by Saccardo (conidia oblong to ovoid, hyaline or olivaceous, 2–3.5 × 0.75–1 μm) was found on the type specimen (Saccardo 1878; van der Aa and Vanev 2002). The Chinese specimen had similar-sized conidia (2–3 × 0.8–1 μm) that were hyaline, oblong to bacilliform without mucilaginous sheaths and apical appendages (Bai 2003, p. 181). This may be an *Asteromella* species.

116. *Phyllosticta physaleos* Saccardo, Michelia, 1: 150, 1878.

→ *Phoma* sp.

Host: *Physalis alkekengi* var. *franchetii* (Solanaceae).

Distribution: Heilongjiang.

Specimen: HMSAU 151.

Notes: The Chinese specimen had conidia that were ellipsoidal, oval, 1-celled, hyaline, 5–7.5 × 2.5–3 μm (Bai 2003, p. 205), which indicates a *Phoma* species.

117. *Phyllosticta pirina* Saccardo, Michelia, 1: 134, 1878.

→ *Phoma pomorum* Thüm.

Hosts: *Pyrus ussuriensis, Pyrus ussuriensis* var. *ovoidea, Pyrus serotina* (Rosaceae).

Distribution: Jilin.

Specimens: HMSAU 739, HMSAU 760, HMSAU 704, HMSAU 738, HMSAU 1913, HMSAU 1133, HMSAU 1172, HMSAU 1173, HMSAU 1176, HMSAU 1175.

Notes: This species has been revised as *Phoma pomorum*, with conidia variable, mostly ovoid or ellipsoidal, 5–7 × 1.5–3 μm (Boerema 1976; Boerema et al. 2004). The Chinese specimens had ovoid, ellipsoidal, 1-celled, hyaline conidia that measured 4–7 × 2–3.5 μm (Bai 2003, p. 176), in good accordance with Boerema’s description.

118. *Phyllosticta pisi* Westendorp, Bull. Ac. Belg. Ser., 12(7):569, 1857.

→ *Phoma* sp.

Host: *Pisum sativum* (Fabaceae).

Distribution: Tibet.

Specimen: HMSAU 2677.

Notes: van der Aa and Vanev (2002) considered this species as *Ascochyta pisi*, which has 2-celled conidia (Chilvers et al. 2009). The Chinese specimen had 1-celled conidia that are hyaline, ellipsoidal, 5–8 × 2–2.5 μm, without mucilaginous sheaths and apical appendages (Bai 2003, p. 124), indicating a *Phoma* species.

119. *Phyllositcta plectranthi* Koval, J. Bot. Acad. Sci. Ukr., 18(2): 76, 1961.

→ *Phoma* sp.

Host: *Plectranthus* sp. (Lamiaceae).

Distribution: Liaoning.

Specimen: HMSAU 2434.

Note: The description of the Chinese specimen (Bai 2003, p. 108) indicates a *Phoma* species with conidia ellipsoidal, 5–7.5 × 2.5–3 μm, without mucilaginous sheaths and apical appendages.

120. *Phyllosticta polygonorum* Saccardo, Michelia, 1: 141, 1878.

→ *Phoma* sp.

Hosts: *Fagopyrum esculentum* (Polygonaceae), *Polygonum orientale, P. platyphyllum*, (Polygonaceae).

Distribution: Heilongjiang, Jilin, Inner Mongolia, Tibet.

Specimens: HMSAU 63, HMSAU 991, HMSAU 2664, HMSAU 1633, HMSAU 2665.

Notes: The Chinese specimens had conidia ovoid, ellipsoidal, hyaline, 4–5.5 × 2–3 μm, without mucilaginous sheaths and apical appendages (Bai 2003, p. 161). This represents a small-spored *Phoma* species.

121. *Phyllosticta populina* Saccardo, Michelia, 1: 155, 1878.

→ *Phoma* sp.

Host: *Populus tomentosa* (Saliceae).

Distribution: Henan.

Specimen: HMSAU 1186.

Notes: Several species including *Mycosphaerella populi, Asteromella* sp. and *Phoma* sp. were observed on the type material of *Phyllosticta populina* (van der Aa and Vanev 2002). The Chinese specimen had conidia that are ellipsoidal, oval, hyaline, 3–6 × 2.5–3 μm (Bai 2003, p. 195), which indicates a *Phoma* species.

122. *Phyllosticta praetervisa* Bubák, Ann. Myc., 2(5):397, 1904.

Host: *Tilia amurensis* (Malvaceae).

Distribution: Jilin.

Specimen: HMSAU 1628.

Notes: In Bubák’s original description, the conidia are short cylindrical, straight, round at both ends, with 2 guttules, 4–5 × 1 μm (Bubák 1904). This species was later treated as a synonym of *Asteromella praetervisa* (Rupprecht 1957). Bai (2003, p. 209) described a fungus with conidiogenous cells ampulliform, 1-celled, hyaline, 4–6 × 2.5–4 μm, conidia bacilliform, enlarged at both ends, 4–6 × 0.7–1 μm. Both the description and sketch showed dumb-bell-shaped conidia. This species is more likely a spermatial state of *Phyllosticta* rather than *Asteromella*, but more characterization is needed to cofirm its identity.

123. *Phyllosticta prinsepiae* G. Z. Lu & J. K. Bai, in Yu et al., Journal of Shenyang Agricultural University, 25: 157, 1994.

Host: *Prinsepia sinensis* (Rosaceae).

Distribution: Liaoning.

Specimen: HMSAU 2046.

Notes: The original literature did not provide a description of this species (Yu et al. 1994); it is therefore an illegal name. The description of *P. prinsepiae* in Bai (2003, p. 183) indicated that the conidia of this species are fusiform or kidney-shaped, with pointed ends, 1-celled, hyaline, 6–8 × 1.5–2.5 μm, without mucilaginous sheaths and apical appendages. This fungus does not belong to *Phyllosticta* but more information is needed to confirm its identity.

124. *Phyllosticta prunicola* (Opiz) Saccardo, Michelia, 1: 157, 1878.

→ *Phoma pomorum* Thüm.

Hosts: *Prunus davidiana, P. humulis, P. mandshurica* (Rosaceae).

Distribution: Jilin.

Specimens: HMSAU 979, HMSAU 1980, HMSAU 1228, HMSAU 737, HMSAU 893.

Notes: The morphology of this record from China (Bai 2003, p. 183) is similar to the *P. pirina* record in leaf spots, pycnidia, and conidia, which most likely represents *Phoma pomorum* species (see note of *P. pirina*).

125. *Phyllosticta rhamni* Westendorp. Bull. Acad. Roy. Belg., 2: 26, 1857.

→ *Phoma* sp.

Host: *Rhamnus ussuriensis* (Rhamnaceae).

Distribution: Liaoning.

Specimens: HMSAU 1979, HMSAU 2077.

Notes: Westendorp mistakenly described the spermatial stage of a rust fungus as *Phyllosticta* (van der Aa and Vanev 2002). In the *Flora Fungorum Sinicorum*, Bai (2003, p. 167) described a coelomycetous fungus, with conidia ovoid or cylindrical, 1-celled, hyaline, 4–7 × 3–4 μm, without mucilaginous sheaths and apical appendages, which indicates a *Phoma* species.

126. *Phyllosticta rhamnicola* Desmazières, Ann. Sci. Nat., 8: 32, 1847.

Host: *Rhamnus dahurica* (Rhamnaceae).

Distribution: Jilin.

Specimen: HMSAU 1211.

Notes: van der Aa and Vanev (2002) indicated that *Mycosphaerella punctiformis* and *Guignardia rhamani* were found on the type specimen of *Phyllosticta rhamnicola*, and the ‘spores’ of *P. rhamnicola* that Desmazières studied were the inner ascomatal cells of *G. rhamani* (van der Aa and Vanev 2002). In the description by Bai (2003, p. 167), the conidia are fusiform, hyaline, round at both side, and constricted in the middle, 4–6 × 1–1.5 μm; while in the illustration, the conidia are dumb-bell-shaped which is incongruent with the description. More information is needed to confirm its identity.

127. *Phyllosticta rhei* Ellis & Everhart, Journ. Myc., 5: 145, 1889.

→ *Phoma* sp.

Host: *Rheum officinale* (Rumiceae).

Distribution: Jilin, Sichuan.

Specimens: HMSAU 992, HMSAU 2640.

Notes: *Phyllosticta rhei* Ellis & Everhart is a later homonym of *P. rhei* Roumeguère. The original description indicates the existence of septa in the conidia (Ellis and Everhart 1889). This species has been reclassified as *Phoma rhei* by Gruyter et al. (2002). The Chinese specimens had smaller conidia than that of *Phoma rhei*, and lack mucilaginous sheaths or apical appendages (Bai 2003, p. 162). This record may represent another *Phoma* species.

128. *Phyllosticta rhododendri* Westendorp, Null. Ac. Bruxell, 18:399, 1851.

→ *Phoma* sp.

Host: *Rhododendron micranthum* (Ericaceae).

Distribution: Liaoning.

Specimen: HMSAU 2044.

Notes: The Chinese specimen had conidia that are 1-celled, hyaline, ellipsoidal or cylindrical, 6–7.5 × 2.5–3 μm, without mucilaginous sheaths and apical appendages (Bai 2003, p. 87). This indicates a *Phoma* species.

129. *Phyllosticta ricini* Rostrup, Bot. Tidsskr., 22: 266, 1899.

→ *Phoma* sp.

Host: *Ricinus communis* (Euphorbiaceae).

Distribution: Jilin.

Specimen: HMSAU 705.

Notes: van der Aa and Vanev (2002) reclassified *Phyllosticta ricini* (conidia 6–7 × 3–4 μm) in *Phoma* species. The Chinese specimen (Bai 2003, p. 94) also pointed to a *Phoma* species but with smaller conidia, 3–5 × 2–3 μm, lacking a mucilaginous sheath and an apical appendage.

130. *Phyllosticta rosicola* Massalongo, Atti d. R. Istit. venedo disc. lett. ed arti, 59: 687, 1900.

→ *Asteromella rosicola* (C. Massal.) H. Ruppr.

Host: *Rosa* sp. (Rosaceae).

Distribution: Inner Mongolia.

Specimen: HMSAU 1591.

Notes: In the original description of *Phyllosticta rosicola*, the conidia are bacilliform, enlarged at both ends, 2.5–4 × 1 μm (Massalongo 1900). In 1959, Rupprecht transferred this species to *Asteromella* (Rupprecht 1959). The Chinese specimen (Bai 2003, p. 188) is morphologically concordant with *Asteromella rosicola*.

131. *Phyllosticta roumeguerii* Saccardo, Michelia, 2: 88, 1880.

→ *Phoma* sp.

Host: *Viburnum sargentii* (Adoxaceae).

Distribution: Liaoning.

Specimen: HMSAU 2070.

Notes: *Phyllosticta roumeguerii* Saccardo and *Phyllosticta roumeguerii* (Saccardo) Allescher have been reclassified as *Phoma exigua* var. *viburni* and *Phomopsis* sp., respectively (van der Aa and Vanev 2002). The Chinese specimen (Bai 2003, p. 66) had conidiogenous cells ampulliform, 1-celled, hyaline, 5–8 × 2 μm, and conidia 1-celled, ellipsoidal, 1–3 guttules, 7–8 × 3–3.5 μm, without mucilaginous sheaths and apical appendages, which indicates a *Phoma* species.

132. *Phyllosticta ruborum* Saccardo, Michelia, 2: 342, 1880.

Host: *Rubus phoenicolasius* (Rosaceae).

Distribution: Shanxi.

Specimen: HMSAU 2710.

Notes: van der Aa and Vanev (2002) reclassified this species as *Phomopsis vepris* (Saccardo) Höhnel. The Chinese specimen had shorter conidia (2–4 × 1–1.5 μm) that lack mucilaginous sheaths and apical appendages (Bai 2003, p. 189). This is not a *Phyllosticta* species, but more information is needed to confirm its identity.

133. *Phyllosticta saccardoi* Thümen. Contr. Myc. Lusit., 28: 48, 1880.

→ *Asteromella* sp.

Host: *Rhododendron micranthum* (Ericaceae).

Distribution: Liaoning.

Specimen: HMSAU 159.

Notes: The Chinese specimen had conidia that are 1-celled, hyaline, ellipsoidal or ovoid, 2–4 × 1–2 μm, without mucilaginous sheaths and apical appendages (Bai 2003, p. 87). This is likely an *Asteromella* species.

134. *Phyllosticta sanguisorbae* Cochrjakov, Not. Syst. Crypt. Inst. Bot. Acad. Sci. USSR, 7: 145, 1951.

→ *Phoma* sp.

Host: *Sanguisorba officinalis* (Rosaceae).

Distribution: Inner Mongolia.

Specimen: HMSAU 1584.

References: Bai (2003). *Flora Fungorum Sinicorum*, vol. 15, pp. 178–179.

Notes: van der Aa and Vanev (2002) indicated that this species could be a *Phomopsis* species with conidia fusiform, 1-celled, biguttulate, 9–12 × 3–4 μm. The Chinese specimen had smaller conidia (5–7 × 2–3 μm) that are oval to ellipsoidal, without mucilaginous sheaths and apical appendages (Bai 2003, p. 178). This is likely a *Phoma* species.

135. *Phyllosticta sanguisorbicola* G. Z. Lu & J. K. Bai, in Yu et al., Journal of Shenyang Agricultural University, 25: 157, 1994.

Hosts: *Sanguisorba officinalis* (Rosaceae), *Agrimonia pilosa* (Rosaceae).

Distribution: Liaoning.

Specimens: HMSAU 2079, HMSAU 2333.

Notes: The original literature did not provide a description (Yu et al. 1994). *Phyllosticta sanguisorbicola* is thus an illegal name. The Chinese specimens had conidia that are bacilliform or kidney-shaped, with round ends, 1-celled, hyaline, 3–4 × 0.5–0.7 μm, without mucilaginous sheaths and apical appendages (Bai 2003, p. 190), typically not a *Phyllosticta* species. More information is needed to confirm its identity.

136. *Phyllosticta schimae* Y.Y. Su & L. Cai, Persoonia, 28: 76, 2012.

Host: *Schima superba* (Theaceae).

Distribution: Zhejiang.

Specimen: HMAS242923 (holotype).

Note: This species is an accepted *Phyllosticta* species with typical morphology and strong phylogenetic inference (Su and Cai 2012; ITS GenBank number: JN692534).

137. *Phyllosticta scrophulariae* Saccardo, Michelia, 1: 159, 1878.

→ *Phoma* sp.

Host: *Scrophularia grayana* (Scrophulariaceae).

Distribution: Jilin.

Specimens: HMSAU 1131, HMSAU 1178, HMSAU 1260.

Notes: van der Aa and Vanev (2002) studied the type material and reclassified this species as a *Phoma* anamorph of *Didymella exigua*. The Chinese specimens had conidia that are ellipsoid to cylindrical, 1-celled, hyaline, 3–7.5 × 1.5–2.5 μm, without mucilaginous sheaths and apical appendages (Bai 2003, p. 200), which indicates a *Phoma* species.

138. *Phyllosticta schimicola* N. Zhou et al. Fungal Biol. X: X, 2015.

Host: *Schima superb* (Theaceae).

Distribution: Jiangxi.

Specimens: HMAS 245575, CGMCC 3.17319, CGMCC 3.17320.

Note: This species is an accepted *Phyllosticta* species with typical morphology and strong phylogenetic inference (Zhou et al. 2015).

139. *Phyllosticta smilacina* Spegazzini, Fg. Arg. Novi v. crit., 315, 1899.

→ *Phoma* sp.

Host: *Smilax campestris* (Smilacaceae).

Distribution: Shanxi.

Specimen: HMSAU 2604.

Notes: van der Aa and Vanev (2002) reclassified this species as *Phomopsis brunneola*, with conidia ellipsoidal, 5–8 × 2–3 μm. The Chinese specimen had similar-sized conidia that are oval to ellipsoidal, without mucilaginous sheaths and apical appendages (Bai 2003, p. 129), and conidiogenous cells that are ampulliform, 1-celled, hyaline, 4–5 × 2–4 μm. This record is more likely a *Phoma* species.

140. *Phyllosticta sonchi* Sacc., Michelia, 1: 141, 1878.

→ *Boeremia exigua* (Desm.) Aveskamp, Gruyter & Verkley.

Host: *Ixeris sonchifolia* (Asteraceae).

Distribution: Liaoning.

Specimen: HMSAU 2351.

Notes: van der Aa and Vanev (2002) reclassified this speices as *Boeremia exigua*. The Chinese specimen had conidia that are 1-celled, hyaline, ellipsoidal, 6–8 × 2.5–3 μm, without mucilaginous sheaths and apical appendages (Bai 2003, p. 78), which is in accordance with *Boeremia exigua*.

141. *Phyllosticta sophoricola* Hollós., Ann. Mus. Nat. Hung., 5: 456, 1907.

→ *Phoma* sp.

Host: *Sophora japonica* (Fabaceae).

Distribution: Liaoning.

Specimen: HMSAU 2041.

Note: The description and brief sketch (Bai 2003, p. 114) indicates a *Phoma* species with conidia oval or ellipsoidal, 5–7 × 3–4 μm, without mucilaginous sheaths and apical appendages.

142. *Phyllosticta sorghina* Sacc., Michelia, 1: 140, 1878.

→ *Phoma sorghina* (Sacc.) Boerema, Dorenb. & Kesteren.

Hosts: *Sorghum vulgaris, S. vulgaris* var. *sudanese, Panicum miliaceum* (Poaceae).

Distribution: Jilin, Mongolia.

Specimens: HMSAU 657, HMSAU 844, HMSAU 706, HMSAU 853, HMSAU 801.

Notes: *Phyllosticta sorghina* has been reclassified as *Phoma sorghina* (Sacc.) Boerema, Dorenb & Kesteren (Boerema 1993). The Chinese specimens had conidia that are ellipsoidal, sometimes curved, 3–6 × 2–2.5 μm, without mucilaginous sheaths and apical appendages (Bai 2003, p. 103), similar to *Phoma sorghina* (Boerema et al. 2004).

143. *Phyllosticta spermoides* Peck, Rep. (Annual) Trustees State Mus. Nat. Hist., New York 40: 58, 1887.

→ *Asteromella* sp.

Host: *Vitis amurensis* (Vitaceae).

Distribution: Liaoning.

Specimen: HMSAU 1984.

Notes: The conidia of the Chinese specimen are bacilliform, 1-celled, hyaline, 3–5 × 0.8–1 μm, without mucilaginous sheaths and apical appendages (Bai 2003, p. 218). These characters are in agreement with *Asteromella* species (van der Aa and Vanev 2002).

144. *Phyllosticta spuriopimpinellae* G.Z. Lu & J.K. Bai, in Yu et al., Journal of Shenyang Agricultural University, 25: 157, 1994.

→ *Phoma* sp.

Host: *Spuriopimpinella brachycarpa* (Apiaceae).

Distribution: Jilin.

Specimen: HMSAU 2038.

Notes: No description was provided when this species was first reported (Yu et al. 1994). In Bai’s description (2003, p. 214), the specimen has conidia that are oval, 1-celled, hyaline, 2.5–4 × 1–1.5 μm, without mucilaginous sheaths and apical appendages. The conidial size is remarkably smaller than typical *Phyllosticta* species. Based on these morphological characters, we regarded that this specimen belong to a small-spored *Phoma* species.

145. *Phyllosticta sterculiae* G. Winter, Bolm soc. Broteriana, Coimbra, Ser. 12: 54, 1884.

→ *Phomopsis* sp.

Host: *Sterculia wallichii* (Malvaceae).

Distribution: Hainan.

Specimen: HMSAU 2209.

Notes: The Chinese specimen had conidiogenous cells that are ampulliform, 1-celled, hyaline, 10–17.5 × 2.5–3.5 μm, and conidia that are fusiform or oval, with pointed ends, 5–7.5 × 2–2.5 μm, without mucilaginous sheaths and apical appendages (Bai 2003, p. 206). The description points to a *Phomopsis* species with α-conidia.

146. *Phyllosticta styracicola* K. Zhang & L. Cai, Mycol. Prog. 12(3), 2012.

Host: *Styrax grandiflorus* (Styracaceae).

Distribution: Yunnan.

Specimen: HMAS 243474 (holotype).

Note: This species is an accepted *Phyllosticta* species with typical morphology and well-inferred phylogeny (Zhang, Zhang, et al. 2013; ITS GenBank number: JX025040).

147. *Phyllosticta syringae* Westendorp, Bull. Acad. R. Sci. Belg., Cl. Sci. 18: 23, 1852.

→ *Phoma* sp.

Host: *Syringa amurensis* (Oleaceae).

Distribution: Liaoning.

Specimen: HMSAU 2234.

Notes: Bresadola (1894) transferred this species to *Ascochyta*. According to the original description of *Ascochyta syringae*, the conidia are 1-septate, but the conidia of the Chinese specimen are aseptate. In addition, both in the original and later description, the conidia of *Ascochyta syringae* are larger than that of the Chinese specimen (8–10 × 3–3.5 μm vs. 4–7.5 × 2–2.5 μm) (Bai 2003, p. 151). The description of this record indicates a *Phoma* species.

148. *Phyllosticta tabaci* Passerini, Atti Soc. Crittogam. Ital. 3: 13, 1881.

→ *Phoma* sp.

Host: *Nicotiana tabacum* (Solanaceae).

Distribution: Jilin.

Specimen: HMSAU 986.

Notes: The conidia of this record are cylindrical, ellipsoidal, 1-celled, hyaline, 4–7 × 2–3 μm, without mucilaginous sheaths and apical appendages (Bai 2003, p. 203). This is likely a *Phoma* species.

149. *Phyllosticta theacearum* van der Aa, Studies in Mycology, 5: 97, 1973.

→ *Phyllosticta capitalensis* Henn.

Host: *Camellia sinensis* (Theaceae).

Distribution: Shandong.

Specimen: MHQAU0192.

Notes: *P. theacearum* has been treated as a synonym of *P. capitalensis* (Baayen et al. 2002). Jin (2011) identified one isolate as *P. theacearum* (Jin 2011). However, no detailed description or DNA sequence was provided except an illustration of conidia. Based on the provided information, we regarded this record as *P. capitalensis.*

150. *Phyllosticta ulmariae* Thümen, Byull. Mosk. Obshch. Ispyt. Prir., boil. 55: 229, 1878.

→ *Phoma* sp.

Hosts: *Spiraea pubescens, S. trilobata* (Rosaceae).

Distribution: Hebei.

Specimens: HMSAU 6635, HMSAU 2612.

Note: This is a *Phoma* species with conidia ellipsoidal, 1-celled, 4–6 × 2.5–3 μm, without mucilaginous sheaths and apical appendages (Bai 2003, p. 185).

151. *Phyllosticta ulmicola* Sacc., Michelia, 1: 158, 1878.

→ *Phoma* sp.

Host: *Ulmus macrocarpa* (Ulmaceae).

Distribution: Liaoning.

Specimen: HMSAU 1532.

Notes: In Saccardo’s original description, the conidia are oblong or ellipsoidal, 6 × 3 μm, hyaline to olivaceous. It has been transferred to *Microsphaeropsis* (van der Aa and Vanev 2002). Bai (2003, p. 211) described a fungus with oval, ellipsoidal, hyaline conidia, 4–6 × 2–3 μm, without mucilaginous sheaths and apical appendages, which should be a *Phoma* species.

152. *Phyllosticta vaccinii* Earle, Bull. Torr. Bot. Club 24: 31, 1897.

→ *Phoma* sp.

Host: *Vaccinium vitis-idaea* (Ericaceae).

Distribution: Inner Mongolia.

Specimen: HMSAU 1627.

References: Bai 2003. *Flora Fungorum Sinicorum*, vol. 15, pp. 90–91.

Notes: *Phyllosticta vaccinii* is an accepted species according to Aa’s examination of type specimen (van der Aa 1973; van der Aa and Vanev 2002), which has recently been epitypified (Zhang, Zhang, et al. 2013). The conidial size of the ex-epitype strain is 7.5–13 × 5–7.5 μm (Zhang, Zhang, et al. 2013). Another accepted *Phyllosticta* species from *Vaccinium* sp. is *P. elongata* (conidial size 12 × 6 μm) (Weidemann et al. 1982). The Chinese specimen (Bai 2003, p. 90) appears to be a *Phoma* species with small conidia (4–8 × 2–4 μm).

153. *Phyllosticta valerianae-tripteris* Unamuno, Mem. R. Soc. Espan. Hist. Nat., 15: 348, 1929.

Host: *Patrinia scabiosaefolia* (Caprifoliaceae).

Distribution: Liaoning.

Specimen: HMSAU 2606.

Notes: This species associated with *Valeriana* species has been reclassified by Gruyter and Noordeloos (1992) as *Phoma valerianae* based on morphology. Recently, De Gruyter et al. 2013) transferred *Phoma valerianae* to *Subplenodomus* based on the phylogeny based on LSU and ITS sequences. The Chinese specimen was associated with *Patrinia* sp. with bacilliform or oblong, 3–4 × 1–1.5 μm conidia without mucilaginous sheaths and apical appendages (Bai 2003, p. 217). More information is needed to determine if this species should be reclassified as *Subplenodomus valerianae.*

154. *Phyllosticta vesicatoria* Thümen, Flora, Regensburg 61: 177, 1878.

→ *Asteromella quercifolii* C. Massal.

Host: *Cyclobalanopsis morii* (Fagaceae).

Distribution: Liaoning, Jilin, Taiwan, Tibet.

Specimens: HMSAU 2636, HMSAU 2747, HMSAU 1463, HMSAU 1530.

Notes: The Chinese specimens had conidia that measured 2–5 × 1–1.5 μm, smaller than any accepted *Phyllosticta* species (Bai 2003, p. 98). There are another two *Phyllosticta* species on *Quercus* sp., i.e. *P. livida* and *P. associate*, with similar conidial sizes 3 × 1 μm and 2–4 × 1 μm respectively. All of these recordes may be *Asteromella quercifolii* (Saccardo 1892).

155. *Phyllosticta vitis* Sacc., Michelia, 1: 135, 1878.

→ *Phoma* sp.

Host: *Vitis amurensis* (Vitaceae).

Distribution: Jilin.

Specimen: HMSAU 2055.

References: Bai (2003). *Flora Fungorum Sinicorum*, vol. 15, pp. 221–222.

Notes: The only accepted *Phyllosticta* species described from *Vitis* was *P. ampecilicida. P. vitis* has been revised as *Phoma negriana* (Thümen 1878; van der Aa and Vanev 2002), with conidia broadly ellipsoidal to oblong, with 2 guttules, 4.5–8.5 × 2–4 μm. The Chinese specimen had conidia that are ovoid, ellipsoidal, 1-celled, hyaline, 3–6 × 1.5–2.5 μm (Bai 2003, p. 221). This is a small-spored *Phoma* species.

156. *Phyllosticta zeae* G. L. Stout, Mycologia, 22: 281, 1930.

→ *Phoma* sp.

Host: *Zea mays* (Poaceae).

Distribution: Tibet.

Specimen: HMSAU 2675.

Notes: *Phyllosticta zeae, P. hispida, P. maydis, P. sorghina* and *P. zeae-maydis* have been recorded associated with *Zea* spp. These species have been reclassified as *Asteromella* sp., *Phoma zeae-maydis, Epicoccum sorghi*, or *Phoma* sp. (van der Aa and Vanev 2002). The Chinese specimen had conidia that were hyaline, 1-celled, ellipsoidal, 4–7 × 2–2.65 μm (Bai 2003, p. 100), which are morphologically similar to that of *P. zeae* G.L. Stout (Stout 1930) and *Phoma zeae-maydis* Sawada (Sawada 1959). Both may belong to *Phoma* (Punithalingam 1990).

157. *Phyllosticta zingiberi* Hori, in Hara, Z. S. B., 438, 1930.

→ *Phoma* sp.

Host: *Zingiber officinale* (Zingiberaceae).

Distribution: Guangdong.

Specimen: HMSAU 2203.

Notes: The Chinese specimen had conidia that are ellipsoidal or oval, 1-celled, hyaline, 5–6 × 2.5–3 μm, without mucilaginouse sheaths and apical appendages (Bai 2003, p. 222). This indicates a *Phoma* species.

158. *Phyllosticta zizyphi* Thümen, Hedwigia 19: 180, 1880.

→ *Microsphaeropsis* sp.

Host: *Ziziphus jujuba* (Rhamnaceae).

Distribution: Liaoning.

Specimen: HMSAU 2040.

Notes: In the original description of *P. zizyphi*, Thümen did not indicate whether the conidia were hyaline or pigmented (Thümen 1880). In van der Aa and Vanev’s (2002) re-description, the conidia of *P. zizyphi* were given as broadly ellipsoidial, 1-celled, hyaline, 4–6 × 2–3 μm. The Chinese specimen had conidia that were oval or ellipsoidal, 1-celled, hyaline or brown, 5–7.5 × 3–4.5 μm, without mucilaginous sheaths and apical appendages (Bai 2003, p. 170). This record was more likely a *Microsphaeropsis* species.

## Disclosure statement

No potential conflict of interest was reported by the authors.

## References

[CIT0001] Aveskamp M, De Gruyter J, Woudenberg J, Verkley G, Crous PW. 2010 Highlights of the Didymellaceae: a polyphasic approach to characterise *Phoma* and related pleosporalean genera. Stud Mycol. 65:1–60. 20502538 10.3114/sim.2010.65.01 PMC2836210

[CIT0002] Baayen R, Bonants P, Verkley G, Carroll G, Aa H, De Weerdt M, Van Brouwershaven I, Schutte G, Maccheroni JW, De Blanco CG, et al. 2002 Nonpathogenic isolates of the citrus black spot fungus, *Guignardia citricarpa*, identified as a cosmopolitan endophyte of woody plants, *G. mangiferae* (*Phyllosticta capitalensis*). Phytopathology. 92:464–477. 18943020 10.1094/PHYTO.2002.92.5.464

[CIT0003] Bai JK 2003 Flora fungorum sinicorum 15. Sphaeropsidales, *Phoma, Phyllosticta*. Beijing: Science Press. Chinese. (in Chinese).

[CIT0004] Batista A, Peres G 1961 Asteromella re-exame de alguns taxa. Memórias da Sociedade Broteriana. 14:28.

[CIT0005] Batista ACV, AF 1952 Monografia das Espécies de *Phyllosticta* em Pernambucu. Boletín da Secretaria de Agricultura Industria e Comercio do Estado de Pernambuco. 19:50.

[CIT0006] Boerema G 1976 The *Phoma* species studied in culture by Dr RWG Dennis. Trans Br Mycol Soc. 67:289–319.

[CIT0007] Boerema G 1993 Contributions towards a monograph of *Phoma* (coelomycetes)-II. Section Peyronellaea. Persoonia. 15:197–221.

[CIT0008] Boerema G, Dorenbosch MMJ 1973 The *Phoma* and *Ascochyta* species described by Wollenweber and Hochapfel in their study on fruit-rotting. Stud Mycol. 3:1–50.

[CIT0009] Boerema G, Gruyter J, Noordeloos M, Hamers MEC 2004 *Phoma* identification manual. Differentiation of specific and infra-specific taxa in culture. Wallingford: CABI Publishing.

[CIT0010] Bresadola G 1894 Fungi aliquot saxonici novi vel critici a cl. W. Krieger lecti. (Contributio III ad Floram Mycol. Saxoniae). Hedwigia. 33:4.

[CIT0011] Bresadola G 1915 Neue Pilze aus Sachsen. Ann Mycol. 13:104–106.

[CIT0012] Brunaud P 1886a Liste des sphaeropsidées trouvées á Saintes et dans les environs. Actes de la Société Linnéenne de Bordeaux. 40:55.

[CIT0013] Brunaud P 1886b Sphaeropsidées nouvelles, rares ou critiques aux environs de Saintes. Rev Mycologique Toulouse. 8:139–142.

[CIT0014] Bubák F 1904 Neue oder kritische Pilze. Ann Mycol. 2:395–401.

[CIT0015] Chi P 1994 Fungal diseases of cultivated medicinal plants in Guangdong province. Guangzhou: Guangdong Science and Technology Press; p. 137–139.

[CIT0016] Chi P, Bai JK, Zhu K 1966 Fungal pathogens on cultivated plants from Jilin. China: Science Press. Chinese.

[CIT0017] Chilvers MI, Rogers JD, Dugan FM, Stewart JE, Chen W, Peever TL 2009 *Didymella pisi* sp. nov., the teleomorph of *Ascochyta pisi*. Mycol Res. 113:391–400. 19116165 10.1016/j.mycres.2008.11.017

[CIT0018] Cooke M 1883 New American fungi. Grevillea. 12:22–33.

[CIT0019] Cooke M 1885 New British fungi. Grevillea. 69:1–14.

[CIT0020] Crous PW, Seifert KA, Castañeda Ruiz RF 1996 Microfungi associated with *Podocarpus* leaf litter in South Africa. S Afr J Bot. 62:89–98.

[CIT0021] De Gruyter J, Woudenberg J, Aveskamp M, Verkley G, Groenewald J, Crous P 2013 Redisposition of *Phoma*-like anamorphs in Pleosporales. Stud Mycol. 75:1–36. 24014897 10.3114/sim0004 PMC3713885

[CIT0022] Delacroix G 1904 Sur quelques champiguons parasites sur les Caféiers. Bulletin de la Société Mycologique de France. 20:10.

[CIT0023] Desmazières J 1847 Quatorzième notice sur les plantes cryptogames récemment découvertes en France. Ann Sci Nat Bot Biol Série. 3:9–37.

[CIT0024] Ellis JB, Dearness J. 1893 New species of Canadian fungi. Can Rec Sci Montreal. 5:267–272.

[CIT0025] Ellis JB, Everhart BM 1889 New and rare species of North American fungi. J Mycol. 5:145–157.

[CIT0026] Ellis JB, Everhart BM 1892 New species of fungi. J Mycol. 7:130–135.

[CIT0027] Ellis JB, Everhart BM 1893 New species of fungi from various localities. Proc Acad Nat Sci Philadelphia. 45:440–466.

[CIT0028] Ellis JB, Everhart BM 1894 New species of fungi from various localities. Proc Acad Nat Sci Philadelphia. 46:322–386.

[CIT0029] Ellis JB, Kellerman WA 1883 New species of North American fungi. Am Nat. 17:1164–1166.

[CIT0030] Farr DF, Rossman AY 2012 Fungal databases, systematic mycology and microbiology laboratory [Internet]. ARS, USDA. [cited 2014 Dec 17]. Available from: http://nt.ars-grin.gov/fungaldatabases/

[CIT0031] Glienke C, Pereira O, Stringari D, Fabris J, Kava-Cordeiro V, Galli-Terasawa L, Cunnington J, Shivas R, Groenewald J, Crous P 2011 Endophytic and pathogenic *Phyllosticta* species, with reference to those associated with Citrus Black Spot. Persoonia Mol Phylogeny Evol Fungi. 26:47–56. 10.3767/003158511X569169 PMC3160796 22025803

[CIT0032] Gruyter J, Boerema G, Aa H 2002 Contributions towards a monograph of *Phoma* (Coelomycetes). VI-2. Section *Phyllostictoides*: outline of its taxa. Persoonia. 18:1–53.

[CIT0033] Gruyter J, Noordeloos M 1992 Ontributions towards a monograph of *Phoma* (Coelomycetes)–I. 1. Section *Phoma*: Taxa with Very Small Conidia in Vitro. Persoonia. 15:71–92.

[CIT0034] Hennings P 1895 Fungi goyazenses. Hedwigia. 34:88–116.

[CIT0035] Hennings P 1905 Fungi japonici VI. Botanische Jahrbücher für Systematik Pflanzengeschichte und Pflanzengeographie. 37:156–166.

[CIT0036] Höhnel F 1912 Fragmente zur Mykologie XIV. Mitteilung (No. 719 bis 792). Sitzungsber Akad Wiss Wien, Mathem Naturwiss Kl, Abt. I. 121:339–424.

[CIT0037] Höhnel FV 1906 Sitzungsberichte der Kaiserlichen Akademie der Wissenschaften in Wien Mathematisch-Naturwissenschaftliche Classe, Abt. Fragmente zur Mykologie. 1:681.

[CIT0038] Hyde KD 1995 Fungi from palms. XX. The genus *Guignardia*. Sydowia. 47:180–198.

[CIT0039] Jin J 2011 Conidial morphology changes in four *Phyllosticta* species. Mycotaxon. 115:401–406.

[CIT0040] Karsten PA 1896 Fragmenta mycologica XLIV. Hedwigia. 35:6.

[CIT0041] Kickx J. 1867 Flore Cryptogamique des Flandres. Paris: Bailliére; p. 1–490.

[CIT0042] Lin X, Huang YJ, Zheng ZH, Su WJ, Qian XM, Shen YM 2010 Endophytes from the pharmaceutical plant, Annona squamosa: isolation, bioactivity, identification and diversity of its polyketide synthase gene. Fungal Divers. 41: 41–51.

[CIT0043] Liu LL, Lu GZ 2007 *Phyllosticta euonymi-japonici* sp. nov., an endophytic fungus isolated from Euonymus japonicus. Mycosystema. 26:171–173.

[CIT0044] Lou B, Xu Y, Sun C, Lou X 2009 First report of leaf blight on duying caused by *Phyllosticta anacardiacearum* in China. Plant Dis. 93:546–546. 10.1094/PDIS-93-5-0546B 30764157

[CIT0045] Massalongo C 1900 De nonnullis speciebus novis micromycetum agri veronensis. Atti dell'Istituto veneto Scienze. 59:683–690.

[CIT0046] Mathur RS 1979 The Coelomycetes of India. Dehradun: Bishen Singh Mahendra Pal Singh; p. 1–460.

[CIT0047] McNeill J, Barrie F, Buck W, Demoulin V, Greuter W, Hawksworth D, Herendeen P, Knapp S, Marhold K, Prado J 2012 International code of nomenclature for algae, fungi, and plants (Melbourne Code). Adopted by the eighteenth International Botanical Congress Melbourne, Australia, July 2011. Regnum Vegetabile. Ruggell: Gantner.

[CIT0048] McPartland J 1994 Cannabis pathogens X: *Phoma, Ascochyta* and *Didymella* species. Mycologia. 86:870–878.

[CIT0049] Miura M 1928 Flora of Manchuria and East Mongolia III. Cryptogams, Fungi. Dalian: South Manch Railwang Co. Japanese.

[CIT0050] Montagne JPFC 1849 Sixièmes Centurie de plantes cellulaires nouvelles, tant indigènes qu’exotiques. Décades III À VI. Annales des Sciences Naturelles Botanique. 11:33–66.

[CIT0051] Motohashi K, Inaba S, Anzai K, Takamatsu S, Nakashima C 2009 Phylogenetic analyses of Japanese species of *Phyllosticta* sensu stricto. Mycoscience. 50:291–302.

[CIT0052] Müller E, Arx JAV 1962 Die Gattungen der didymosporen Pyrenomyceten. Beiträge zur Kryptogamenflora der Schweiz. 11:1–922.

[CIT0053] Muntanola-Cvetkovic M, Mihaljcevic M, Petrov M 1981 On the identity of the causative agent of a serious Phomopsis-Diaporthe disease in sunflower plants. Nova Hedwigia. 34:417–435.

[CIT0054] Naito N 1940 Notes on some new or noteworthy fungi of Japan. Mem of the Coll of Agric, Kyoto Univ. 47:45–52.

[CIT0055] Passerini G. 1881 Di alcune crittogame osservate sul tabacco. Atti della Socieà Crittogamologica Italiana. 3:13–16.

[CIT0056] Passerini 1888 Atti Accad. Naz. Lincei, Rendiconti Adunanze Solenni, Ser. 4:66.

[CIT0057] Persoon CH 1818 Traité sur les champignons comestibles: contenant l’indication des espèces nuisibles: précédé d’une introduction a l’histoire des champignons. Paris: Chez Belin-Leprieur Libraire; p. 1–276.

[CIT0058] Petrak F 1914 Beiträge zur Pilzflora von Mähren und Österr.-Schlesien. Ann Mycol. 12:9.

[CIT0059] Petrak F 1934 Mykologische notizen XII. Ann Mycol. 32:317–447.

[CIT0060] Pu J, Xie Y, Zhang X, Qi Y, Zhang C, Liu X 2008 Preinfection behaviour of *Phyllosticta musarum* on banana leaves. Australas Plant Pathol. 37:60–64.

[CIT0061] Punithalingam E 1975 Some new species and combinations in *Phomopsis*. Trans Br Mycol Soc. 64:427–435.

[CIT0062] Punithalingam E 1990 CMI descriptions of fungi and bacteria: Set 102, nos. 1011–1020. Mycopathologia. 112:39–63.

[CIT0063] Ramakrishnan T 1942 A leaf spot disease of Zingiber officinale caused by *Phyllosticta zingiberi* n. sp. Proc Indian Acad Sci Sect B. 15:167–171.

[CIT0064] Rupprecht H 1957 Beitrage zur Kenntnis der Fungi imperfecti. Sydowia. 11:1–6.

[CIT0065] Rupprecht H 1959 Beiträge zur Kenntnis der Fungi imperfecti. III. Sydowia. 13:12.

[CIT0066] Saccardo PA 1878 Fungi Veneti novi vel critici vel mycologiae Venetae addendi. Series VII. Michelia. 1:133–221.

[CIT0067] Saccardo PA 1884 Sylloge Fungorum: Sylloge Sphaeropsidearum et Melanconiearum. Sylloge Fungorum. 3: 1–840.

[CIT0068] Saccardo PA 1891 Fungi abysinici a cl. O. Penzig Collecti. Malpighia. 5:274–287.

[CIT0069] Saccardo PA 1892 Sylloge Fungorum Omnium Hucusque Cognitorum 10. Sumptibus auctoris, Typis seminarii, Patavii, Italy; Pavia; p. 1–964.

[CIT0070] Saccardo PA 1895 Supplementum universale, Pars. III. Sylloge Fungorum. 11: 1–753.

[CIT0071] Saccardo PA 1896 Fungi aliquot brasilienses phyllogeni. Bulletin de la Société Royale de Botanique. 35:127–132.

[CIT0072] Saccardo PA 1899 Supplementum Universale, Pars IV. Sylloge Fungorum. 14: 1–1316.

[CIT0073] Saccardo PA 1902 Supplementum Universale, Pars V. Sylloge Fungorum. 16: 1–1291.

[CIT0074] Saccardo PA 1903 Notae mycologicae. Series III. Ann Mycol. 1:24–29.

[CIT0075] Saccardo PA 1906 Sylloge Fungorum Omnium Hucusque Cognitorum. Pavia, Italy. 18: 1–838.

[CIT0076] Saccardo PA 1915 Notae mycologicae. Series XIX. Annales Mycologici. 13:115–138.

[CIT0077] Saccardo PA, Trotter A 1931 Supplementum Universale, Pars X. Myxomycetae, Myxobacteriae, Deuteromycetae, Mycelia sterilia. Sylloge Fungorum. 25:1–1093.

[CIT0078] Sawada K 1959 Descriptive catalogue of Taiwan (Formosan) fungi. Part XI. Spec Publ Coll Agric Natl Taiwan Univ. 8:1–268.

[CIT0079] Shear CL 1907 Cranberry diseases U.S. Dept Agr Bur Plant Indus Bul. 110:1–64.

[CIT0080] Stout G 1930 New fungi found on the Indian corn plant in Illinois. Mycologia. 22:271–287.

[CIT0081] Su Y, Cai L 2012 Polyphasic characterisation of three new *Phyllosticta* spp. Persoonia Mol Phylogeny Evol Fungi. 28:76–84. 10.3767/003158512X645334 PMC3409417 23105155

[CIT0082] Sutton BC 1980 The Coelomycetes. Fungi imperfecti with pycnidia, acervuli and stromata. Kew: Commonwealth Mycological Institute; p. 1–696.

[CIT0083] Sydow H 1916 Fungi Indiae Orientalis Pars V. Ann Mycol. 4:424–445.

[CIT0084] Sydow P 1897 Beiträge zur Kenntnis der Pilzflora der Mark Brandenburg. I. Hedwigia Beiblätter. 36:157–164.

[CIT0085] Sydow P 1899 Fungi Natalensis. Hedwigia. 38:130–134.

[CIT0086] Tai FL 1979 Sylloge Fungorum Sinicorum. Beijing: Science Press; p. 1–1527. Chinese.

[CIT0087] Teng SC 1963 Fungi of China. Beijing: Science Press; p. 1–808. Chinese.

[CIT0088] Thümen F 1878 Die Pilze des Weinstockes. Wien: Wilhelm Braumüller; p. 1–225.

[CIT0089] Thümen F 1880 Contributiones ad floram mycologicam Lusitanicam. Ser. II (Instituto de Coimbra, 1879, XXVII) (Schluss). Hedwigia. 19:6.

[CIT0090] van der Aa HA 1973 Studies in *Phyllosticta*. Stud Mycol. 5:1–110.

[CIT0091] van der Aa HA, Boerema G, Gruyter J 2000 Contributions towards a monograph of *Phoma* (Coelomycetes) VI-1: section *Phyllostictoides*: characteristics and nomenclature of its type species Phoma exigua. Persoonia. 17:435–456.

[CIT0092] van der Aa HA, Vanev S 2002 A revision of the species described in *Phyllosticta*. Utrecht, The Netherlands: Centraalbureau voor Schimmelcultures; p. 510.

[CIT0093] Vuillemin MP 1888 L’ascospora beijerinckii et la maladie des cerisiers. Journal de Botanique. 2:255–259.

[CIT0094] Wang X, Chen G, Huang F, Zhang J, Hyde KD, Li H 2012 Phyllosticta species associated with citrus diseases in China. Fung Divers. 52:209–224.

[CIT0095] Weidemann G, Boone D, Burdsall H Jr 1982 Taxonomy of *Phyllosticta vaccinii* (Coelomycetes) and a new name for the true anamorph of *Botryosphaeria vaccinii* (Dothideales, Dothioraceae). Mycologia. 74:59–65.

[CIT0096] Westendorp GD 1851 Sur quelques cryptogames inedites ou nouvelles pour la Flore Belge. Notice deuxieme. Bulletin de l'Académie Royale des Sciences de Belgique Classe des Sciences. 18:384–416.

[CIT0097] Wikee S, Lombard L, Crous PW, Nakashima C, Motohashi K, Chukeatirote E, Alias SA, McKenzie EHC, Hyde KD 2013 Phyllosticta capitalensis, a widespread endophyte of plants. Fung Divers. 60:91–105.

[CIT0098] Wikee S, Udayanga D, Crous PW, Chukeatirote E, McKenzie EHC, Bahkali AH, Dai DQ, Hyde KD 2011 *Phyllosticta*—an overview of current status of species recognition. Fung Divers. 51:43–61.

[CIT0099] Wu S-P, Liu Y-X, Yuan J, Wang Y, Hyde KD, Liu Z-Y 2014 Phyllosticta species from banana (Musa sp.) in Chongqing and Guizhou Provinces, China. Phytotaxa. 188:135–144.

[CIT0100] Wulandari N, To-Anun C, Hyde K, Duong L, De Gruyter J, Meffert J, Groenewald J, Crous P 2009 *Phyllosticta citriasiana* sp. nov., the cause of Citrus tan spot of *Citrus maxima* in Asia. Fung Divers. 34:23–39.

[CIT0101] Wulandari NF, To-Anun C, Lei C, Abd-Elsalam KA, Hyde KD 2010 *Guignardia/Phyllosticta* species on banana. Cryptogam Mycol. 31:403–418.

[CIT0102] Xing X, Chen J, Xu M, Lin W, Guo S 2011 Fungal endophytes associated with *Sonneratia* (Sonneratiaceae) mangrove plants on the south coast of China. For Pathol. 41:334–340.

[CIT0103] Young E 1915 Studies in Porto Rican parasitic fungi. I. Mycologia. 7:143–150.

[CIT0104] Yu L, Lu GZ, Liu WC, Bai JK 1994 Studies on the taxonomy of genera of *Phoma* and *Phyllosticta* in North-eastern China. J Shenyang Agric Univ. 25:153–158.

[CIT0105] Zhang K, Su Y-Y, Cai L 2013 Morphological and phylogenetic characterisation of two new species of *Phyllosticta* from China. Mycoll Prog. 12:547–556.

[CIT0106] Zhang K, Zhang N, Cai L 2013 Typification and phylogenetic study of *Phyllosticta ampelicida* and *P. vaccinii*. Mycologia. 105:1030–1042. 23709479 10.3852/12-392

[CIT0107] Zhou N, Chen Q, Carroll G, Zhang N, Shivas RG, Cai L 2015 Polyphasic characterization of four new plant pathogenic *Phyllosticta* species from China, Japan and the United States. Fung Biol. doi: 10.1016/j.funbio.2014.08.006. 25937069

